# New Biotechnological Tools for the Genetic Improvement of Major Woody Fruit Species

**DOI:** 10.3389/fpls.2017.01418

**Published:** 2017-08-15

**Authors:** Cecilia Limera, Silvia Sabbadini, Jeremy B. Sweet, Bruno Mezzetti

**Affiliations:** ^1^Department of Agricultural, Food and Environmental Sciences, Università Politecnica delle Marche Ancona, Italy; ^2^J. T. Environmental Consultants Ltd Cambridge, United Kingdom

**Keywords:** plant breeding, RNA interference (RNAi), trans-grafting, cisgenesis/intragenesis, Crispr/Cas9, genome editing, EU biosafety regulations

## Abstract

The improvement of woody fruit species by traditional plant breeding techniques has several limitations mainly caused by their high degree of heterozygosity, the length of their juvenile phase and auto-incompatibility. The development of new biotechnological tools (NBTs), such as RNA interference (RNAi), trans-grafting, cisgenesis/intragenesis, and genome editing tools, like zinc-finger and CRISPR/Cas9, has introduced the possibility of more precise and faster genetic modifications of plants. This aspect is of particular importance for the introduction or modification of specific traits in woody fruit species while maintaining unchanged general characteristics of a selected cultivar. Moreover, some of these new tools give the possibility to obtain transgene-free modified fruit tree genomes, which should increase consumer's acceptance. Over the decades biotechnological tools have undergone rapid development and there is a continuous addition of new and valuable techniques for plant breeders. This makes it possible to create desirable woody fruit varieties in a fast and more efficient way to meet the demand for sustainable agricultural productivity. Although, NBTs have a common goal i.e., precise, fast, and efficient crop improvement, individually they are markedly different in approach and characteristics from each other. In this review we describe in detail their mechanisms and applications for the improvement of fruit trees and consider the relationship between these biotechnological tools and the EU biosafety regulations applied to the plants and products obtained through these techniques.

## Introduction

Conventional breeding for genetic improvement of woody fruit crops is a slow and difficult process, with drawbacks caused by high heterozygosity, extended juvenile periods, and auto-incompatibility (Petri and Burgos, 2005; Rai and Shekhawat, 2014). Furthermore, improvement of woody fruit species using conventional breeding methods is a long-term process because of their long generation time. New biotechnological tools (NBTs) including genetic engineering methods can promote the prompt insertion of important genes into the genome of commercial woody fruit cultivars, thus resulting in more efficient and reliable genetic improvement (Lusser et al., 2012) of clonal propagated plants, maintaining high stability of the major traits of the clone. The introduction of recombinant DNA technology paved the way for an immense potential in the field of plant biotechnology. In order to attain food security and to guarantee nutritional quality, NBTs for generating genetically modified (GM) plants with useful agronomic and quality traits are already of high significance for many crops (Datta, 2013; Qaim and Kouser, 2013).

Genetic engineering in plants has been in practice for more than three decades. Direct transformation methods (Biolistic) and indirect methods (*Agrobacterium tumefaciens*-mediated transformation), developed decades ago, have been the primary strategies of heterologous DNA introduction into plants (Chilton et al., 1977; Gelvin, 2003; Altpeter et al., 2005). All genetically modified crops commercially grown, including woody fruit species, were produced using one of these methods (Parisi et al., 2016). Often the ability to obtain fruit tree plants with new traits or mutations by genetic engineering or by NBTs depends on the existence of a well-established *in vitro* regeneration protocol, which depends on the genotype and the type of starting plant tissue used (Wang et al., 2011; Rai and Shekhawat, 2014; Saporta et al., 2017). Furthermore, it is more advisable from an agronomic point of view to *in vitro* regenerate a new fruit tree plant from mature tissues, due to the high degree of heterozygosity, which characterize the majority of these species (Cervera et al., 1998; Pérez-Jiménez et al., 2012). In this sense relevant progress have been made during the last two decades for some difficult-to-transform woody species, such as peach or grapevine genotypes, in which efficient protocols for the regeneration of adventitious shoots have been developed starting from adult tissues (Mezzetti et al., 2002; Pérez-Jiménez et al., 2012; Sabbadini et al., 2015). Introduction of one or more new genes or regulatory elements using genetic engineering techniques, directly manipulates the genome of an organism in order to express or silence specific traits (Tzfira and Citovsky, 2006; Mittler and Blumwald, 2010; Rai and Shekhawat, 2014). Transgenic approaches having global impact are aimed mainly at the production of crops with new resistance genes against pests and diseases, or herbicide tolerance, such as Monsanto's roundup ready crops (soya, maize, and cotton; Funke et al., 2006; Lombardo et al., 2016; Parisi et al., 2016), and plants with enhanced desirable qualities and nutritional levels, such as the golden rice with an increased vitamin A content (Paine et al., 2005; Bhullar and Gruissem, 2013; Pérez-Massot et al., 2013; Zhu et al., 2013; Giuliano, 2017).

In woody fruit species, the use of conventional plant breeding techniques such as traditional mutation, translocation breeding, and intergeneric crosses, is very limiting due to the non-specific approaches often leading to mutation of thousands of untargeted nucleotides instead of the single desired one or the transfer of a large part of the genome instead of a single gene (Hartung and Schiemann, 2014). It is for this reason that gene transfer, site-specific integration, and specific regulation of gene expression are crucial advancements in plant biotechnology (Datta, 2013). In this review we describe the mechanisms of the more advanced biotechnological techniques and their application in woody fruit species improvement.

NBTs used for modifying an existing DNA sequence in a plant, comprise of insertion/deletion and gene replacement, or stable silencing of a gene or promoter sequence. In this category we consider techniques such as RNA interference (RNAi), cisgenesis/intragenesis, trans-grafting, and gene editing techniques including zinc finger nucleases (ZFNs) as well as clustered regularly interspaced short palindromic repeats/CRISPR-associated protein 9 (CRISPR/Cas9 system), to introduce new traits into a host plant genome. All these technologies have been successfully applied in different crops, but there are still limited applications in woody fruit species.

## Cisgenesis and intragenesis

**Figure 1:**
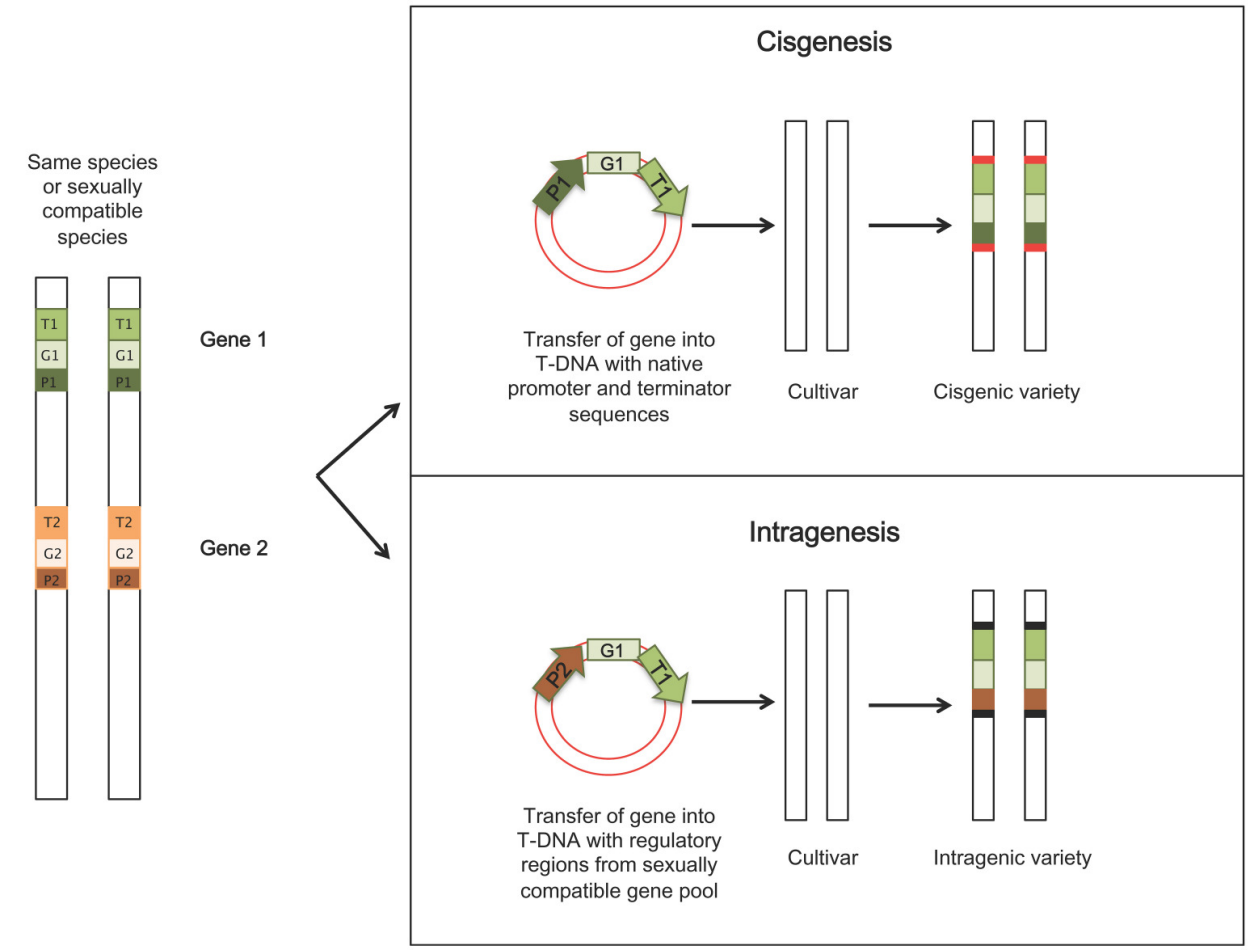
Illustration of principles and procedures of obtaining cisgenic and intragenic crops. In cisgenesis, the new trait is derived from a sexually compatible species and it is transferred to the recipient as it is, including the *Agrobacterium*-derived T-DNA borders; in intragenesis, the gene construct is a hybrid of different components from different genes within the same species or sexually compatible species. Red boxes: *Agrobacterium*-derived T-DNA borders; black boxes: borders belonging to sexually compatible DNA pool (P-DNA borders), when using *Agrobacterium*-mediated transformation. P, promoter; G, engineered gene; T, terminator.

The term cisgenesis was introduced by Schouten et al. (2006a), defining it as the genetic modification of plants using genes that originate only from the species itself or from a species that can be crossed conventionally with this species. The added gene is an extra copy to the existing genome and is a natural variant, which includes its introns, flanking native promoter and terminator in normal sense orientation (Lusser and Davies, 2013). In intragenesis, the introduced genetic element (intragene) originates from the same species or a species from a sexually compatible gene pool. The intragenes are considered hybrid genes since they can be driven by different promoter or terminator regions of different genes and loci (Rommens, 2007). The inserted DNA sequence will form a new arrangement of genetic elements leading to a modified functional version compared to the starting genome (Conner et al., 2007). Furthermore, in intragenic plants, when using *Agrobacterium*-mediated transformation as strategy to insert the new trait, plant-derived transfer DNA (P-DNA) borders sequences from the sexually compatible DNA pool are used in order to avoid accidental insertion of vector sequences (Rommens, 2004). Thus, it is possible to obtain transformed plants which do not contain any foreign DNA. These approaches avoid the potential for “linkage drag” (the transfer of other undesirable genes along with the gene of interest), associated with classical introgression in conventional breeding (Jacobsen and Schouten, 2007). Whole genomic sequencing studies are providing information on the cisgenes that can be used for genetic improvement of specific crops, but in many cases the availability of cisgenic promoters and efficient marker genes are limited. An illustration of the two techniques is shown in Figure 1.

Cisgenesis/intragenesis has been applied in different woody fruit species including apples. Fruit breeders are developing solutions for the various diseases affecting apples, including fire blight disease caused by *Erwinia amylovora*. Kost et al. (2015) recently developed a cisgenic apple line C44.4.146 from a fire blight susceptible cultivar “Gala Galaxy” using the cisgene FB_MR5 from wild apple *Malus* × *robusta* 5 (*Mr5*), which confers resistance to fire blight (Peil et al., 2007). After elimination of the selectable markers through heat-induced recombinase, both PCR and Southern blot analysis did not detect any transgenes. The transformed line C44.4.146 carried just the cisgene FB_MR5 and its native regulatory sequences (Kost et al., 2015). Cisgenesis and intragenesis have also been successfully applied to induce resistance to other diseases in both apple and other woody fruit tree and vines, as shown in Table 1.

**Table 1:** Applications of cisgenesis and intragenesis in woody fruit species.

**Plant species**	**Name of gene**	**Source**	**Trait**	**Achievement**	**References**
Apple (*Malus domestica*)	*HcrVf*2	Apple (*Malus domestica*)	Resistance to Apple scab (*Venturia inaequalis*)	80% reduction in fungal infection of the cisgenic lines compared with the scab-susceptible 'Gala'	Joshi et al., 2011; Vanblaere et al., 2011, 2014;
Apple (*Malus domestica*)	*Rvi6*	Apple (*Malus floribunda* 821)	Resistance to Apple scab (*Venturia inaequalis*)	Cisgenic plants had similar resistance to the *M. floribunda* control	Krens et al., 2015
Apple (*Malus* × *domestica* Borkh)	*Rvi6*	Apple (*Malus floribunda* 821)	Resistance to Apple scab (*Venturia inaequalis*) strain 104 (Race 1)	Two cisgenic lines resistant to (*Venturia inaequalis*) strain 104 (Race 1)	Wurdig et al., 2015
Apple (*Malus* × *domestica* Borkh)	*HcrVf2*	Apple cv Gala	Resistance to Apple *Rvi6* scab	Cisgenic lines containing the *HcrVf2* gene	Gessler et al., 2014
Apple (*Malus domestica*)	*FB_MR5*	Apple cv Gala Galaxy	Resistance to fire blight (*Erwinia amylovora*)	Cisgenic line C44.4.146, expressing the cisgene *FB_MR5*, with lower disease symptoms when inoculated with *Erwinia amylovora*	Kost et al., 2015
Grapevine (*Vitis vinifera* L.)	*VVTL*-1	Grapevine (*Vitis vinifera*)	Resistance to Powdery mildew (*Erysiphe necator*)	Cisgenic plants showed a delay in powdery mildew disease development and decreased severity of black rot *(Guignardia bidwellii*) during field tests	Dhekney et al., 2011
Grapefruit (*Citrus paradisi*)	*C. clementina*-derived T-DNA-like region	*Citrus clementina*	Development of “foreign DNA-free” intra-/cisgenic citrus cultivars	Transformation efficiency in “Duncan” grapefruit was ~0.67%	An et al., 2013

## RNA interference (RNAi)

The first discovery of the silencing phenomenon in plants was made in 1990, by scientists trying to deepen the purple color of petunias through the overexpression of *Chalcone synthase* gene. Contrary to their expectations, the flowers became white indicating that the gene had been turned off (Napoli et al., 1990; Metzlaff et al., 1997). The suppression of endogenous gene expression through the introduction of a homologous sequence into the genome was referred to as “co-suppression” in petunia (Campbell and Choy, 2005), later correlated to the phenomenon of post-transcriptional gene silencing (PTGS).

RNAi is an endogenous cellular process that occurs naturally to “turn off” unwanted or harmful specific nucleic sequences, or to regulate gene expression before translation (Baum et al., 2007; De Alba et al., 2013). RNAi has been discovered and studied in many organisms such as fungi, animals, and ciliates (Romano and Macino, 1992; Fire et al., 1998; Billmyre et al., 2013; Scott et al., 2013), and has more recently been studied in plants (Matzke et al., 2001; Baulcombe, 2004; Ipsaro and Joshua-Tor, 2015).

RNAi refers to a complex of molecular mechanisms, which have the main function of gene expression inhibition or suppression, activated by the presence of double-stranded RNA molecules (dsRNAs; Voinnet, 2008; Parent and Vaucheret, 2012). The discovery of this process led to the possibility of creating custom “knock-downs” of gene activity. In both plants and animals it has been shown that RNAi utilizes the dsRNAs as trigger molecules that detect homologous mRNAs, whose transcription is negatively regulated (Almeida and Allshire, 2005; Ketting, 2011; Ipsaro and Joshua-Tor, 2015). Consequently, RNA silencing has emerged as a preferred method for gene targeting in fungi (Nakayashiki, 2005; Salame et al., 2011), insects (Scott et al., 2013), bacteria (Escobar et al., 2001; Navarro et al., 2006), viruses (Baulcombe, 2004; Ding, 2010), and plants (Brodersen and Voinnet, 2006; Frizzi and Huang, 2010). Presently, there are several routes of gene silencing identified in plants, these include: PTGS (Vaucheret et al., 2001; Borges and Martienssen, 2015), transcriptional gene silencing (TGS; Vaucheret and Fagard, 2001; De Alba et al., 2013), and microRNA silencing (miRNA; Bartel, 2004; Jonas and Izaurralde, 2015). All these pathways rely on the presence of dsRNA molecules of different sizes, which are processed into the plant cell by specific protein families, i.e., Dicer or Dicer-like (DCL), Argonaute (AGO), and RNA-dependent RNA polymerases (RDRs; Molnar et al., 2011).

**Figure 2:**
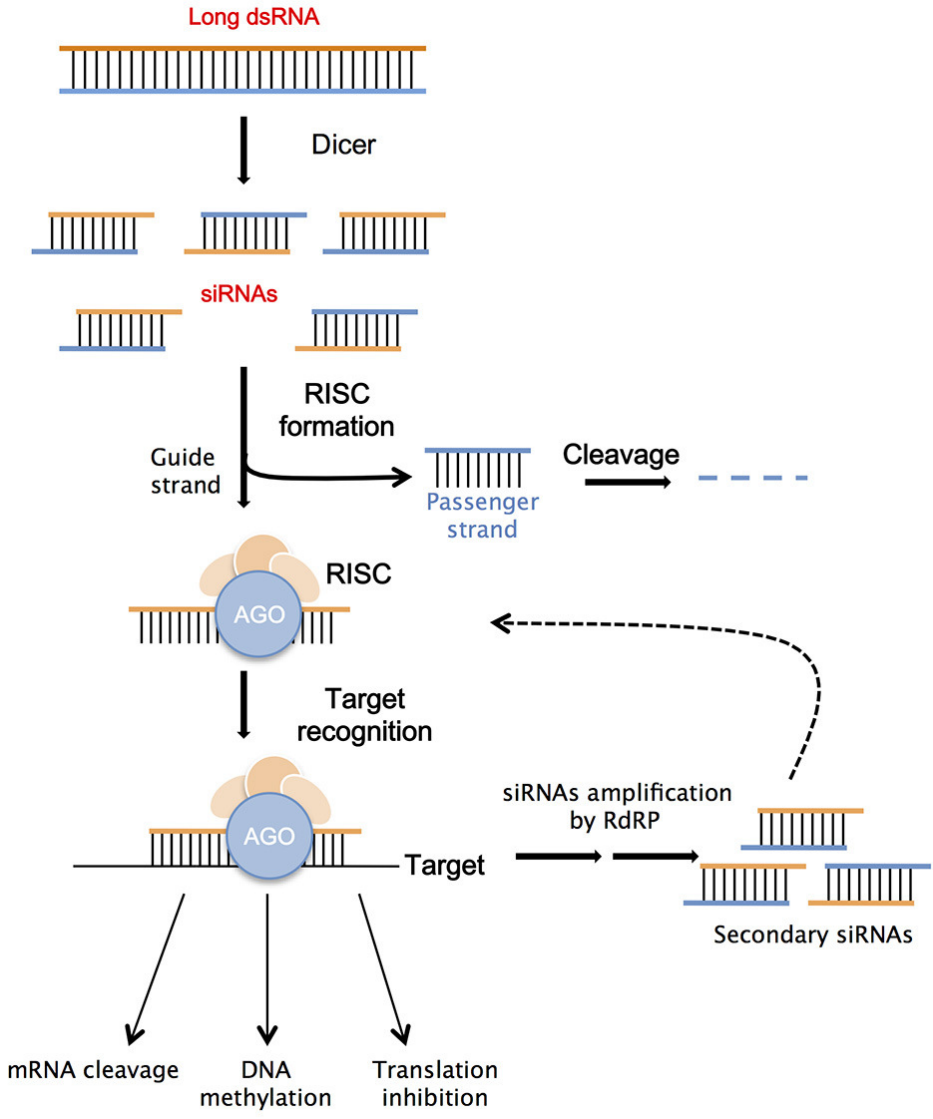
Schematic illustration of RNAi mechanism. Double stranded RNA (dsRNA) molecule binds to a Dicer protein, which cleaves it into small interfering RNAs (siRNAs); these siRNAs bind to an Argonaute (AGO) protein, part of the RNA-Induced Silencing Complex (RISC). The RISC separates the siRNAs into two strands: the passenger strand (blue) is degraded while the guide strand (orange) serves as a search probe, which links RISC to complementary RNA targets. After this recognition target's expression can be regulated through several different mechanisms. In plants, the silencing signal can be perpetuated by the action of the RNA-dependent RNA polymerase (RdRP).

Long dsRNAs constitute the precursors for siRNA molecules (siRNAs) production, thanks to specific Dicer enzymes, whose action determines their final length. DCL1 is responsible for the creation of miRNAs, which originate in the plant cell's nucleus from endogenous precursors characterized by a stem-loop with imperfect double-strand structure (Voinnet, 2009). Twenty-two to twenty-four nucleotide long siRNAs are produced into the cell's cytoplasm by the action of DCL2, which cleaves exogenous long dsRNAs originated from viral intermediates or transgenes (Meister and Tuschl, 2004; Carthew and Sontheimer, 2009). DCL3 cleaves long dsRNAs transcribed in the nucleus by the plant RNA polymerase Pol IV, to produce 24-nt long siRNAs with the main function of heterochromatin modifications (Qi and Hannon, 2005; Brosnan and Voinnet, 2011; Matzke and Mosher, 2014). Finally, DCL4 is responsible for the production of 21-nt secondary siRNAs involved in cell-to-cell silencing signaling (Dunoyer et al., 2005). The siRNAs produced through the various RNAi pathways, are unwound into the passenger and the guide strand; the latter is bound to Argonaute (AGO) proteins to form the nucleus of the RNA-Induced Silencing Complex (RISC). It has been observed that AGO 1, AGO2, AGO 7, and AGO10 bind to siRNAs to induce degradation of the complementary mRNAs or to inhibit translation (Brodersen et al., 2008). The association of siRNAs with other types of AGO proteins (4, 6, or 9) can activate the TGS mechanism, which has the main role of inducing epigenetic changes by chromatin modifications and histone methylation through the RNA-directed DNA methylation (RdDM) pathway (Xie et al., 2004; Brosnan and Voinnet, 2011; Matzke and Mosher, 2014). The silencing signal can be amplified through the action of an RNA-dependent RNA polymerase (RdRP), which helps the perpetuation of the silencing response by the synthesis of secondary siRNAs (Figure 2). This pool of secondary molecules can induce systemic silencing in the plant (Meister and Tuschl, 2004; Carthew and Sontheimer, 2009). The silencing signal, constituted mainly by 21 and 24 nt long siRNAs, has the ability to move from cell to cell and systematically over long distances inside the plant, with the principal role of defense from invasive nucleic acids or to induce epigenetic modification (Molnar et al., 2011). In particular, the local cell to cell movement of siRNAs takes place through the plasmodesmata or by apoplastic transfer; while, the systemic movement occurs through the vascular system, generally starting from a photosynthetic source to end in a sucrose sink (Melnyk et al., 2011).

The most recent discovery in RNA silencing is the cross—talk occurring between kingdoms (Knip et al., 2014). Studies carried out on plants and their fungal pathogens in the laboratory indicate that both parties can move RNAs back and forth into each other's cells. Fungal microbes utilize RNAi to enhance their spread whereas, plants seem to use this mechanism to encounter infection by these pathogens. In both cases desired outcome is achieved through the same molecular process of RNA interference, which interrupts gene expression through target messenger RNA degradation (Cheng et al., 2015; Grens, 2017).

In plants, RNA silencing affects the regulation of endogenous gene expression, and it is also an evolutionary conserved mechanism that serves as host defense against viruses (Ding and Voinnet, 2007; Carbonell and Carrington, 2015). RNAi has been mainly applied in woody fruit species to induce pathogen resistance. Pathogen derived resistance (PDR) is based on the expression of pathogen genetic elements (Sanford and Johnston, 1985; Baulcombe, 1996) which has led to various forms of plant virus resistance (Simón-Mateo and García, 2011). One of the first applications of this approach was the induction of virus resistance through the introduction of gene constructs expressing viral sequences, such as coat protein (CP), movement protein, and replicase (Abel et al., 1986; Baulcombe, 1996; Gottula and Fuchs, 2009). Subsequently several studies showed that the virus resistant phenotypes were often based on the induction of an RNA-mediated mechanism and not on a protein-mediated resistance (English et al., 1996; Hannon, 2002). In early 1990s' a regeneration and transformation protocol to genetically engineer papaya for PRSV resistance, the most widespread and damaging virus disease of papaya, was developed. The objective was to introduce a gene construct that codes for a chimeric coat protein (CP) containing 17 amino acids of the Cucumber mosaic virus and the N terminus of the CP gene of *PRSV* HA 5-1. The inhibition of PRSV obtained in one of the transgenic papaya lines showed an RNA-mediated resistance (Gonsalves, 1998, 2006). Another case of virus resistance in fruit trees, based on RNA-mediated mechanism of PDR, is represented by the transgenic plum clone Honeysweet resistant to sharka or plum pox disease (Scorza et al., 2013). Sharka is considered one of the most devastating diseases in stone fruits and is caused by the *plum pox virus* (PPV; Cambra et al., 2006). PPV-resistant plum was obtained using hypocotyl slices as starting explants, which were transformed with the coat protein gene of *plum pox virus* (PPV-CP). The integration of the engineered *CP* gene was confirmed in five transgenic lines (Scorza et al., 1994). After years of testing, it was shown that the high resistance to PPV of the transgenic clone C5 displayed the typical characteristics of PTGS mechanism (Scorza et al., 2001); 24-nt long siRNAs were detected in the resistant clone when infected by PPV, which were considered responsible for the Honeysweet PPV resistance. dsRNA was demonstrated to be the triggering molecule of RNA silencing, and virus-resistant plants were obtained through a second generation technology based on the introduction of inverted repeat RNA (hairpin RNA) or intron—hairpin-RNA (ihpRNA) constructs into cells, which are able to provide efficient virus resistance by eliciting PTGS in the host plant (Smith et al., 2000; Collinge et al., 2010; Khalid et al., 2017). Hily et al. (2007) demonstrated for the first time that ihpRNA technology could be exploited to obtain virus resistance in *Prunus domestica*. Table 2 shows applications of RNAi technique in other woody fruit species performed over the years with the purpose of inducing disease resistance, post-harvest quality improvement as well as gene functional studies.

**Table 2:** Applications of RNA interference in woody fruit species.

**Plant species**	**Name of gene**	**Source**	**Trait**	**Achievement**	**References**
Papaya (*Carica papaya*)	*PRSV-*CP	*Papaya ringspot virus* (PRSV)	Resistance to PRSV	Transgenic papaya resistant to *Papaya ringspot virus* (PRSV)	Gonsalves, 1998, 2006
Plum (*Prunus domestica* L**.)**	*PPV*-CP	*plum pox virus* (PPV)	Resistance to Sharka (PPV)	Transgenic plum clone Honeysweet resistant to sharka disease	Scorza et al., 1994, 2001, 2013
Sweet orange (*Citrus sinensis*)	*CPsV*-CP	*Citrus psorosis virus* (CPsV*)*	Resistance to CPsV	Transgenic sweet orange plants resistant to CPsV	Reyes et al., 2011
Grapefruit (*Citrus paradisi*)	*CTV*	*Citrus tristeza virus* (CTV)	Resistance to CTV	Transgenic grapefruit lines resistant to CTV	Febres et al., 2008
Apple (*Malus domestica*)	*MdMLO*19	Apple (*Malus domestica*)	Resistance to powdery mildew (*Podosphaera leucotricha*)	Transgenic apple lines resistant to powdery mildew	Pessina et al., 2016
Apple (*Malus domestica*)	*iaaM* and *ipt*	*Agrobacterium tumefaciens*	Resistance to crown gall formation	Transgenic apple lines resistant to crown gall formation on tree roots	Viss et al., 2003
Pear (*P. communis* L.)	*MdTFL1*	Apple (*Malus domestica*)	Early flowering induction	Silencing of *PcTFL1-1* and *PcTFL1-2* genes in transgenic pear with consequent early flowering phenotype	Freiman et al., 2012
Apple (*Malus domestica*)	*MdGA*20-ox	Apple (*Malus domestica*)	The obtainment of dwarf varieties	Transgenic apple lines with reduced height, shorter internode length, and higher number of nodes	Zhao et al., 2016
Apple (*Malus domestica*)	*MdAG*-like genes: *MdMADS*15 and *MdMADS*22	Apple (*Malus domestica*)	The reduction of fertility and the increase of Floral Attractiveness	Trees with polypetalous flowers. Reduced male and female fertility of flowers	Klocko et al., 2016
Apple (*Malus domestica*)	*Endo-polygalacturonase*1 (*PG*1)	Apple (*Malus domestica*)	Improve post-harvest fruit quality	Increased post-harvest fruit quality	Atkinson et al., 2012

## Trans-grafting technique

This technique focuses mainly on grafting, a horticultural technique that has been practized for centuries to improve the quality and yield of fruit crops (Melnyk and Meyerowitz, 2015). The method pairs two autonomous genotypes selected individually for their rooting ability and fruiting characteristics. They are grafted together in order to combine their superior traits in the scion and the rootstock. It has been extensively used to improve crop quality and productivity and also to propagate woody perennial crops like fruits, and ornamental plants (Mudge et al., 2009). The rootstock can alter the phenotype of the scion, for example by reducing its vigor and encouraging more fruit set, but the rootstock and scion retain their genetic integrity, in that the grafted tissues are joined but their genetic materials do not mix. Other tissue grafting techniques include applications ranging from plant breeding to animal organ transplants.

Traditionally grafting is used to improve disease resistance, in particular against soil-borne fungi and bacteria, and growth characteristics such as rooting ability, nutrient, and water acquisition (Haroldsen et al., 2012b). Trans grafting is a method which combines traditional grafting practices with genetic modification of plants. The technique involves grafting of a non-genetically modified scion onto a genetically modified rootstock. The scion acquires benefits and traits conferred by transgenes in the rootstock, but the end products, such as fruits, do not contain the transgene and hence are not genetically modified (Schaart and Visser, 2009; Haroldsen et al., 2012a; Lemgo et al., 2013).

The movement of RNA molecules through the vascular system from the rootstock to the scion is at the basis of trans-grafting technique (Mallory et al., 2003; Lucas and Lee, 2004; Stegemann and Bock, 2009; Haroldsen et al., 2012a). Higher plants function as integrated organisms due to long-distance transport of signaling molecules through phloem, which has emerged as a major communication mechanism that ensures synchronized differentiation and supply of nutrients (McGarry and Kragler, 2013). For example, Lang et al. (1977) demonstrated the mobility of *florigen* in tobacco plants for the promotion and inhibition of flower formation in a neutral-day plant by grafting with a short-day plant and a long-day plant. This discovery helped in understanding the regulation and coordination of tissue formation by plants, making it possible to manipulate flowering time and meiosis thereby controlling crop breeding processes. Recent studies have shown that phloem transports some specific RNA molecules to coordinate organ development (Palauqui et al., 1997; Melnyk et al., 2011; Nazim and Kim, 2013). Research of functional analyses of phloem shows that over 15% of the transcripts are signal transduction related (Omid et al., 2007). If RNAi-based rootstocks can efficiently transfer the silencing molecules to non-transformed scions, then RNAi can be applied to obtain virus resistant transgenic plants (Schaart and Visser, 2009; Lemgo et al., 2013). Recent researches show that siRNA molecules derived from hairpin gene constructs can spread between cells and systemically over long distances (i.e., 1.2 m above the graft union) in woody plants (Haroldsen et al., 2012a; Zhao and Song, 2014), and can induce direct epigenetic modifications at the DNA level of the recipient cells in *Arabodopsis thaliana* (Molnar et al., 2010). In addition, microRNAs and trans-acting siRNAs have been associated with the transmission of silencing signals systemically via phloem and from cell to cell through the plasmodesmata (Nazim and Kim, 2013; Zhao and Song, 2014). Compatibility is important for scion-rootstock interactions for the downward flow of photosynthesis products and upward movement of water and mineral nutrients (Aloni et al., 2010), as well as for the transmission of the RNAi silencing signal into the scion, and initiation of systemic silencing.

Genetically modified rootstocks have the potential to boost production of standard, non-genetically modified fruit varieties while avoiding concerns about transgene flow and exogenous protein production that occur in other types of transformed fruits (Haroldsen et al., 2012a; Song et al., 2013). Considering the applications of this technique (Table 3), it is evident that the use of genetically modified rootstocks for grafting might be the answer to disease control in many woody fruit species through the production of healthy non-genetically modified fruits. These fruits should not need the level of biosafety scrutiny normally required for traditional genetically modified plants.

**Table 3:** Applications of trans-grafting in woody fruit species.

**Plant species**	**Name of gene**	**Source**	**Trait**	**Achievement**	**References**
Apple (*Malus domestica*)	*rolB*	Apple (*Malus domestica*)	Control of scion vigor and reduce plant height	*rolB* transgenic rootstocks significantly reduced vegetative growth including tree height regardless of scion cultivar	Welander and Zhu, 2000; Smolka et al., 2010
Grapevine (*Vitis vinifera* L.)	*Shiva*-1 lytic peptide	Grapevine (*Vitis vinifera*)	To control Pierce's disease (PD) (*Xylella fastidiosa*)	Non-transgenic scion resistant to PD	Dutt et al., 2007
Sweet cherry (*Prunus avium*)	*PNRSV*	*Prunus necrotic ringspot virus* (PNRSV)	Resistance to PNRSV in non-transgenic scions	Non-transgenic scion of sweet cherry grafted onto the transgenic rootstock showed resistance to PNRSV caused by the transportation (rootstock-to-scion) of hpRNA-derived siRNAs	Song et al., 2013; Zhao and Song, 2014

## Gene editing techniques

**Figure 3:**
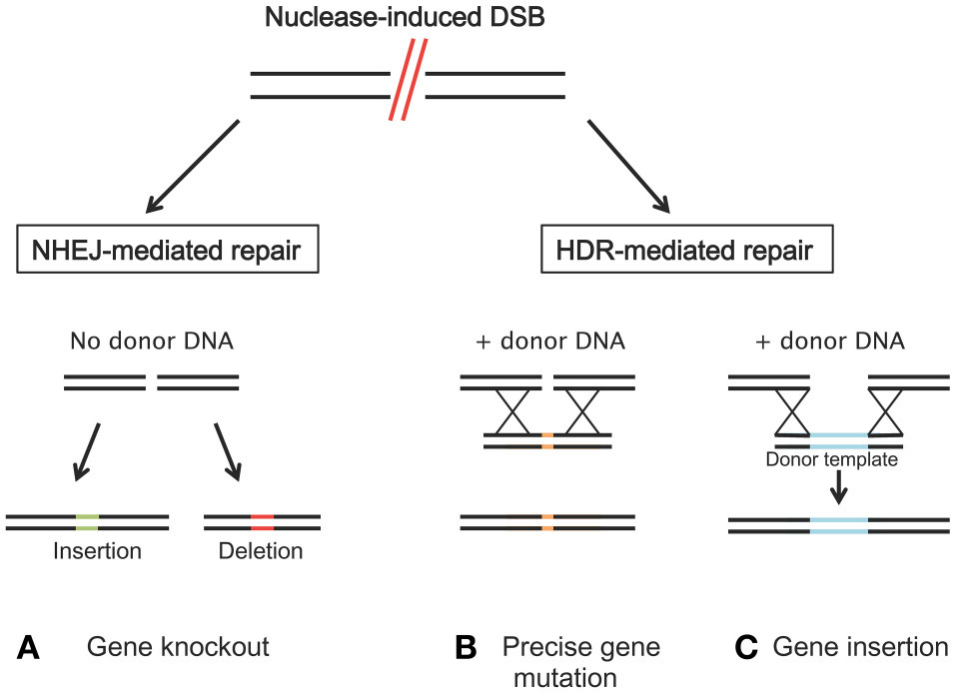
Induced double strand breaks (DSBs) of the target DNA by nucleases can be repaired by either non-homologous end-joining (NHEJ) or homology directed repair (HDR). **(A)** NHEJ usually leads to gene knockout by the insertion (green) or deletion (red) of random base pairs. **(B)** If a donor template, that shares regions of homology to the sequence next to the DSB is available, HDR can introduce precise gene modification or **(C)** specific nucleotide/gene insertion.

A decade ago, a new approach emerged that makes it possible for researchers to manipulate almost any gene in different cell types and organisms. This fundamental technique, commonly referred to as “genome editing” integrates, deletes, and/or mutates genes of interest. Engineered nucleases composed of sequence-specific DNA-binding domains attached to non-specific DNA cleavage modules are at the heart of genome editing techniques (Urnov et al., 2010). The potential to manipulate genetic information in a precise manner and obtain improved plants not only provides the opportunity to create novel phenotypes but also enables biological mechanism and gene function studies. The ability to cleave specific DNA sequences and to induce different DNA repair mechanisms allows for a range of genomic modifications from single-nucleotide mutations to large sequence deletions, rearrangements and/or integrations (Figure 3; Curtin et al., 2012).

### Zinc finger nuclease (ZFN) and TALE nucleases (TALENs)

**Figure 4:**
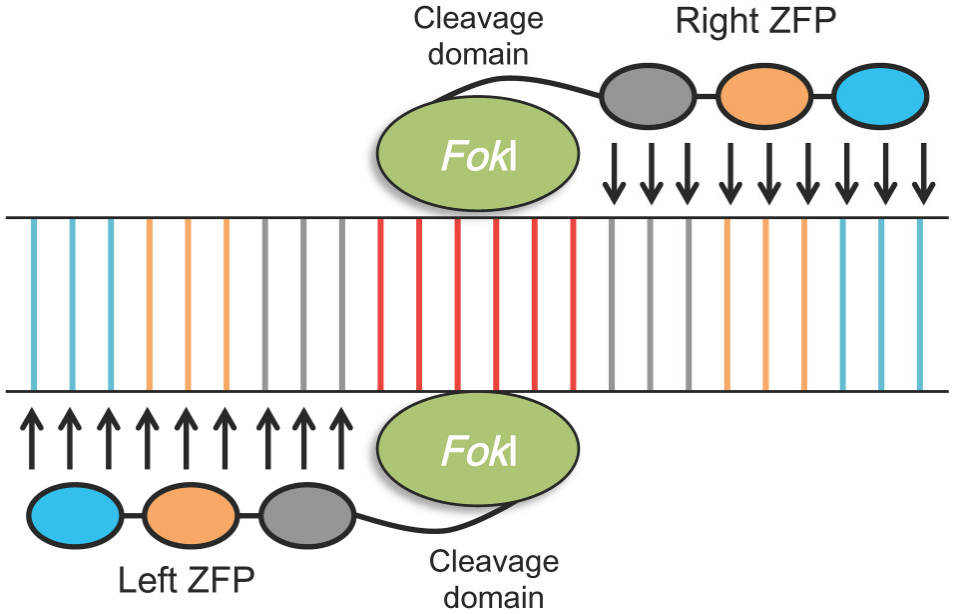
Schematic illustration of zinc-finger nuclease (ZFN) structure and mechanism of inducing double strand breaks (DBSs) on its target. The target site of the ZFN is recognized by the “left” and “right” monomers consisting of a tandem array of three to six engineered zinc finger proteins (ZFPs) (three are shown here); single engineered ZFP can recognize a nucleotide triplet (shown in different colors). Each ZNF is linked to a nuclease domain from the *Fok*I restriction enzyme. Recognition of the target sequence by the left and right ZFPs results in dimerization of the *Fok*I nuclease; DNA cleavage takes place along the spacer sequence (usually 6 bp long, shown in red) between the two ZFP recognition sites.

After the discovery of the functional principles of the Cys2-His2 zinc finger (ZF) motifs, and of truncated transcription activator-like effector (TALE) domains, a first generation of engineered endonucleases (EENs), zinc-finger nucleases (ZFNs), and TALE nucleases (TALENs), were developed (Pabo et al., 2001; Wood et al., 2011). Both ZFNs and TALENs can easily target DNA cleavage and they have been adopted as tools for making directed genetic changes, as they also facilitate the rearrangement of their DNA-binding domain. They are designed and utilized for generating double-strand breaks (DSBs) at almost any specific genomic position to enable genome editing (Urnov et al., 2010; Gaj et al., 2013). DSBs are subsequently exposed to cellular DNA repair mechanisms, which include error-prone non-homologous end joining (NHEJ) and homology-directed repair (HDR), that lead to high frequencies of both targeted mutagenesis, genome editing and targeted gene replacement/integration (Carroll, 2011; Petolino, 2015; Figure 3). ZFNs and TALENs are composed of two proteins, one that is necessary for DNA targeting and binding, which can be engineered to target specific DNA sequences, and a fused nuclease, usually *Fok*I, which cuts the target DNA in a non-specific manner; the two proteins are artificially connected by a peptide linker (Hartung and Schiemann, 2014). Zinc finger proteins (ZFPs) bind to their DNA targets as monomers (fingers), each of which recognizes 3 bp of DNA. ZFNs of 3–6 monomers can be used to target specific DNA sequences of about 9–18-bases long as shown in Figure 4. In the case where a longer target sequence needs to be edited, longer ZFN recognition sequences (24–36 bp) are required for binding to achieve a higher specificity level and reduction of off-site cleavage (Miller et al., 2007; Petolino, 2015). In contrast with ZFNs, TALENs are characterized by DNA binding domains composed by repeats of 33–35 amino acids, each of which is able to recognize a single DNA base pair. This represents an advantage in terms of design flexibility (Gaj et al., 2013). The target specifity of TALENs relies on the presence of two amino acids called repeat-variable di-residues (RVDs; Deng et al., 2012). As with ZFNs, TALENs can also act as modular repeats to target adjoining DNA sequences.

To our knowledge, only ZFNs have been applied in woody fruit species, in particular in apple and fig in a targeted mutagenesis experiment. Protocol optimisation for the use of ZFNs in apple and fig trees was developed by Peer et al. (2015). In this study, the ability of QQR-ZFN to repair a mutated *uidA* gene, which encodes for a non-functional GUS reporter protein, was explored. Both transient and stable transformation studies were carried out in fig and apple *in vitro* tissues. Whole plants with repaired *uidA* gene were regenerated; GUS assay results showed an overall gene editing efficiency of 80–100% per leaf explant in fig and 10–40% per leaf explant in apple.

### Clustered regularly interspaced short palindromic repeats (CRISPR) and CRISPR-associated protein (Cas9)

**Figure 5:**
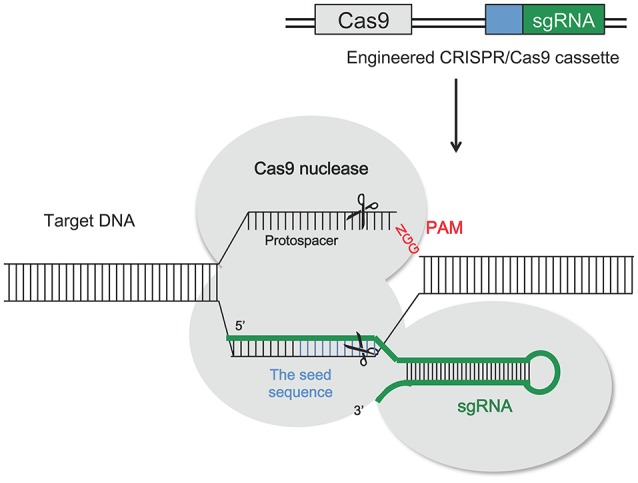
Schematic illustration of the CRISPR/Cas9 system structure and the principle of CRISPR/Cas9-mediated genomic modifications. Cas9 can be reprogrammed to induce the cleavage of specific DNA sequences by the production of a synthetic guide RNA (sgRNA). It contains a region (seed sequence, usually 8–12 bp long, shown in blue) complementary to the target DNA on the genomic loci that mediates the binding of the Cas9 protein. The cleavage site stays 3 bp upstream of the protospacer adjacent motif (PAM, NGG; shown in red), which is required for the cleavage of the target DNA sequence. Induced DSBs of the target DNA are repaired by either NHEJ or HDR, producing gene mutations that include nucleotide insertion, deletion or substitution around the cleavage sites (see Figure 3).

ZFNs (Townsend et al., 2009; Carroll, 2011) and transcription activator-like effector nucleases (TALENs; Boch et al., 2009; Moscou and Bogdanove, 2009) were the main genome editing tools until recently. Due to the difficulties related to the creation of flexible DNA-binding proteins, new methods of targeting such as CRISPR/Cas9 significantly simplified the creation of custom nucleases. Engineered nucleases have been designed as tools for genome editing as efficient genetic engineering methods to target and cleave DNA sequences at specific locations in the genome of both plants and animals (Bortesi and Fischer, 2015; Osakabe and Osakabe, 2015). CRISPR/Cas9 systems are an integrated part of the adaptive immune system of bacteria (*Streptococcus pyogenes*) and archaea (Bhaya et al., 2011; Jinek et al., 2012; Barrangou, 2015; Bortesi and Fischer, 2015), which protects them from invading nucleic acids such as viruses. This adaptive immunity is provided through silencing of the invading nucleic acids using CRISPR RNAs (crRNAs) and the Cas9 nucleases (Horvath and Barrangou, 2010). Cas9 gene in bacterial genomes was found to be closely linked with short, highly homologous sequences arranged in tandem repeats with a varying size between 21 and 37 bp interspaced with non-homologous spacer sequences (Jansen et al., 2002; Bhaya et al., 2011). Immunity is acquired by integrating into the genome short fragments of DNA from the invading organism (spacers), between two adjacent repeats at the proximal end of a CRISPR locus (Bortesi and Fischer, 2015). The spacer sequences determine the target to be cleaved by the endonuclease. The CRISPR arrays, which include the spacers, are transcribed during every encounter with invading DNA and are processed into 40 bp-long small interfering CRISPR RNAs (crRNAs), which combine with the trans-activating CRISPR RNA (tracrRNA) to activate and guide the Cas9 nuclease in cleaving the invading nucleic acid (Bortesi and Fischer, 2015). Target recognition is dependent on the “protospacer adjacent motif” (PAM) which is downstream of the target sequence and usually has the sequence 5′-NGG-3′ adjacent to the 3′ end of the 20 bp target (Jinek et al., 2012; Bortesi and Fischer, 2015). The application of this natural immune system to plant genome editing needs the creation of a single guide RNA molecule (sgRNA), obtained by fusing the 3′ end of the crRNA to the 5′ end of the tracrRNA. In this way Cas9 is reprogrammed to induce the cleavage of specific DNA sequences. A schematic illustration of the CRISPR/Cas9 mechanism is shown in Figure 5.

In comparison to ZFNs and TALENs which are larger in size and require a pair of proteins to recognize target DNA strands for DSBs induction, CRISPR/Cas9 is smaller in size making it easier to co-deliver multiple sgRNAs with Cas9 to the cell, so that the simultaneous editing of more than one target sequence is achievable in a process called “multiplex gene editing” (Cong et al., 2013). CRISPR/Cas9 system specificity is determined by the guide sequence of sgRNA complementing that of the target DNA. For efficient target cleavage by Cas9 to occur, there must be perfect base pairing between the last 8–12 bases of the guide sequence, called the “seed sequence,” and the complementary region of the target DNA (i.e., the region proximal to the 5′ end of the PAM; Jiang et al., 2013). Cas9 acts alone to bind and cleave the DNA target in a sequence-dependent manner (Anders et al., 2014; Nishimasu et al., 2014). The CRISPR/Cas9 system is used widely for genome editing because of its simplicity, design flexibility and high efficiency, which is easily applicable in laboratories. It is also the least expensive and most user-friendly of the three genome-editing tools (Nagamangala et al., 2015). CRISPR/Cas9 system has been applied in a number of woody fruit species to determine the possibility of precise gene mutations induction (Table 4). The most recent application of CRISPR/Cas9 system in inducing disease resistance in woody fruit species has been done in citrus by Peng et al. (2017). This genome editing technique was applied to increase resistance against Citrus canker, caused by *Xanthomonas citri* subsp. *citri* (*Xcc*), a deadly disease threatening the citrus industry worldwide (Stover et al., 2014). Peng et al. (2017) targeted the modification of the EBE_PthA4_ (effector binding element) of the susceptibility gene *LATERAL ORGAN BOUNDARIES* 1 (*CsLOB*1) promoter (Hu et al., 2014) in Wanjincheng orange. During infection, the main Xcc transcription activator-like (TAL) effector, PthA4, connects with the EBE_PthA4_ effector present on the promoter of *CsLOB1* susceptibility gene, activating its expression and inducing Citrus canker development (Hu et al., 2014). Results showed that editing of *CsLOB1* gene promoter was sufficient to increase the resistance of Wanjincheng orange against Citrus canker. In addition high levels of resistance to this disease were also induced by deletion of the entire EBE_PthA4_ sequence from both *CsLOB1* alleles (Peng et al., 2017).

**Table 4:** Applications of CRISPR/Cas9 in woody fruit species.

**Plant species**	**Name of gene**	**Source**	**Trait**	**Achievements**	**References**
Sweet orange (*Citrus sinensis*)	*CsPDS*	Sweet orange (*Citrus sinensis*)	Induce mutation in *CsPDS* sequence	*CsPDS* gene was mutated at the target site in treated sweet orange leaves	Jia and Wang, 2014
Apple (*Malus domestica*)	Phytoene desaturase (*PDS*)	Apple (*Malus domestica*)	Induce mutation in *PDS* sequence	Clear and partial albino phenotypes were observed in 31.8% of regenerated plantlets, and bi-allelic mutations in apple *PDS* were confirmed by DNA sequencing	Nishitani et al., 2016
*Citrus sinensis* Osbeck	EBE_PthA4_ of the of the *CsLOB1* promoter	Wanjincheng orange (*Citrus sinensis* Osbeck)	Mutation in the EBE_PthA4_effector to induce citrus canker resistance	High rate of resistance to citrus canker by mutate the EBE_PthA4_effector	Peng et al., 2017
Grape (*Vitis vinifera* L., cv. Neo Muscat)	*Vitis vinifera phytoene desaturase (VvPDS)* gene	grape (*Vitis vinifera* L., cv. Neo Muscat)	Induce mutation in *VvPDS* gene sequence	Regenerated plants with albino leaves were obtained. DNA sequencing confirmed mutation at the target site of *VvPDS* gene in regenerated grape plants	Nakajima et al., 2017
Grapevine (*Vitis vinifera* L.)	L-idonate dehydrogenase gene (*IdnDH*)	Grapevine (*Vitis vinifera* L.)	Genome editing and targeted gene mutation	100% mutation frequency in the transgenic cell mass (CM) as well as corresponding regenerated plants expressing sgRNA1/Cas9	Ren et al., 2016
Grapevine (*Vitis vinifera* L.) and apple (*Malus domestica*) protoplasts	Grape gene *MLO*-7 and the apple genes *DIPM*-1, 2, and 4	Grapevine (*Vitis vinifera* L.) and apple (*Malus domestica*)	Resistance to powdery mildew in grape and resistance to fire blight disease in apple	Efficient targeted mutagenesis in the protoplasts of both grape *MLO*-7 and the apple *DIPM*-1, 2, and 4	Malnoy et al., 2016

### Oligonucleotide-directed mutagenesis (ODM)

Oligonucleotide-directed mutagenesis **(**ODM) is a gene-editing technique aimed to introduce a new mutation in the plant genome by replacing one or few base pairs (Lusser et al., 2011). This site-specific mutation occurs by the introduction of chemically synthesized DNA oligonucleotides or also chimeric DNA-RNA fragments of 20–100 nucleotides, which are delivered into the plant cells mainly by biolistic methods or electroporation of protoplasts (Breyer et al., 2009; Sauer et al., 2016). The introduced oligonucleotide hybridizes with a complementary predetermined DNA sequence in the plant genome, leading to the creation of a mismatch of one or two base pairs corresponding to the non-homologous nucleotides. This mechanism induces the cell's natural repair machinery to recognize the single base mismatch and to correct it. As a result, the desired specific change in the plant's genome is produced and the oligonucleotide is subsequently degraded by the cell (Schaart et al., 2016). ODM represents theoretically an improved technique over conventional breeding and traditional mutagenesis techniques, due to its controlled and accurate manner of action, through which random mutations are prevented and no recombinant DNA introduction is involved. Thus the final product produced is often similar to conventionally bred or traditional mutagenesis products (Breyer et al., 2009). To our knowledge there are no examples of ODM applications to woody fruit species and few data exist for other plant species (maize, rice, tobacco, and wheat; Zhu et al., 2000; Iida and Terada, 2005; Dong et al., 2006), probably due to some drawbacks, such as the low mutation efficiency and frequency, difficulties in the regeneration of mutated plants and the introduction of spontaneous somatic mutations which hide the changes introduced by ODM (Ruiter et al., 2003; Sauer et al., 2016).

## Biosafety considerations for the application of NBTs in fruit trees

Advancements in agricultural technology offer new products and new solutions toward a sustainable future. However, these come with new concerns and new issues to address. Biosafety risk assessment principles, procedures, and policies have been adopted for ensuring the environmental and personal safety of genetically modified organisms (GMOs). Genetically modified crops present numerous biosafety issues and plant breeders are required to demonstrate the safety of their product before releasing GM crops into the environment for commercial purposes. NBTs have been developed to enable more precise genetic modifications of plants compared to conventional and some early genetic modification methods. However, it is still not clear whether crops obtained using some of these techniques should be classified and regulated as GMOs or the same as a product from traditional breeding or mutagenesis. In particular at EU level there is a lack of clear regulation concerning the use of these new techniques, while the world wide scientific community suggests that the evaluation of plants obtained by NBTs should focus on the changes made to the plant itself and on the final products obtained (Hartung and Schiemann, 2014; Sprink et al., 2016). Therefore, the products from the application of these technologies should be assessed with a simplified procedure mostly addressed to consider whether the induced genomic modifications are within the normal genetic variability of the species.

In terms of risk assessment, one of the main concerns related to GM plants is linked to the production of a new protein in the modified plant and their possible off-target effects. This concern should be avoided by the applications of the cisgenic approach since all components are obtained from the same species or from a sexually compatible species, and thus do not produce a novel protein in the recipient genetically modified plant, and therefore do not provide different outcomes compared to traditional breeding (Podevin et al., 2012).

The EFSA GMO Panel considers that “the Guidance for risk assessment of food and feed from genetically modified plants and the Guidance on the environmental risk assessment of genetically modified plants are applicable for the evaluation of food and feed products derived from cisgenic and intragenic plants/crops. It can be envisaged that on a case-by-case basis lesser amounts of event specific data are needed for the risk assessment” [European Food Safety Authority (EFSA), 2012].

From these considerations, the assessment of a new cisgenic plant in the EU could be reduced to the genomic characterization of the product so that molecular studies can confirm the absence of heterologous DNA sequences and products (proteins and enzymes), and no additional environmental and food safety risk assessment might be needed (Schouten et al., 2006b). Regulatory authorities in some North and South American countries consider that cisgenic plants and intragenic plants not containing antibiotic resistance markers do not need to be considered as other types of GM plants and so would not require regulation (Waltz, 2011).

The full genome sequences now available for many crops offer enormous possibilities to identify useful genes/promoters directly from the same species to be transferred to improve the commercial cultivars. However, the transformation approach can be limited by the availability of efficient selectable markers suitable to replace the commonly used antibiotic or herbicide resistance markers. To solve this problem, progress is being made in developing reporter genes derived from the large class of myeloblastosis (MYB) transcription factors involved in anthocyanin pigment activation in plant species (Elomaa et al., 2003). This approach has been applied in grapevines by Kandel et al. (2016), who compared the grapevine-derived *Vv*MybA1 transcription factor with existing reporter genes *gfp* and *gus*. The *MybA1* reporter gene was found to be suitable for identification of gene expression events at the cell culture level (Kandel et al., 2016). MYB markers can be identified for each plant species, but it is not always easy to develop efficient regeneration systems that allow the application of only a reporter gene without the application of selectable markers.

RNAi-based GM plants regulate the expression of specific genes, determined by the production of dsRNA molecules, without the production of new homologous and heterologous proteins/enzymes. However, impacts on the RNAi gene target and possible off-target effects also need to be considered. Several approaches have been developed to silence plant endogenous genes (e.g., silencing genes for fruit ripening; Atkinson et al., 2012) or the *MLO* gene for mildew resistance (Pessina et al., 2016). In these cases, if there are no other transgenic sequences inserted, the plant could be considered a cisgenic plant and the risk assessment could be reduced to molecular characterization of the event and of the target gene silenced. In case of RNAi systems to induce resistance to other organisms interacting with the plant (e.g., virus, fungi, bacteria, and insects) a study of possible off-target gene silencing through the dsRNAs produced by the plant, in both target and non-target organisms, should be investigated.

The application of GM techniques and, in particular, RNAi technology on rootstocks for producing non-GM grafted scions, are offering new important opportunities for fruit and other vegetatively propagated plants. In the scion, including the fruit, there is no presence of homologous and heterologous proteins but only of small fragments of RNA. The scion does not contain transgenic DNA or novel proteins therefore; the main concerns should be related only to the off-target effects on non-target organisms and sequences by RNAi. An additional benefit of the trans-grafting approach is the absence of pollen or seed dispersal of transgenic material from the non-genetically modified scion.

Biosafety concerns affecting genetically engineered fruit trees also include off-target mutations. Taking into consideration the gene editing approaches (CRISPR/Cas9 system, ZFN, TALEN's, and ODM) it has been demonstrated that their application can result in mutations similar to those that occur in nature or by use of traditional breeding techniques, with the important difference that these new technologies act in a much more specific way (Curtin et al., 2011; Tzfira et al., 2012; Hartung and Schiemann, 2014; Ren et al., 2016). In the CRISPR/Cas9 technique, bioinformatics tools are used in the designing of sgRNAs to identify both target and off-target sites. These tools choose the gene regions for sgRNA creation on the basis of specific sequence characteristics such as size (usually 21–23 bases) and specific nucleotide constitution, thus minimizing the possibility of off-target mutations (Brazelton et al., 2016). One of the final products of this novel gene editing approach can be considered as a point mutation as the double strand break is made in a very precise manner in that it is un-differentiable from natural mutation. For this reason the scientific community has supported the view that CRISPR/Cas9-edited plants should not be classified as GMOs unless they contain transgenic elements. However, the method commonly used to introduce this genome editing system is with *Agrobacterium-*mediated transformation. In this case, the first product obtained is a GMO, for this reason some authorities suggest for the product's regulation. The transgenic complex can be eliminated only by an F_1_ segregation from mutant F_0_ after selfing or back-crossing with the wild type. Segregants in the offspring no longer contain the transgenes, or foreign DNA in their genome. These F_1_ mutants should only differ from the wild type by a small deletion in the target gene, and so they are usually indistinguishable from those arising spontaneously or through mutation breeding (Jones, 2015). In homozygous (seed propagated plants), where selfing- can be used, the F_1_ mutant maintains all the traits of the original cultivar. In heterozygous plants, fruit trees and many other vegetatively propagated plants, back-crossing is required and the gene-edited offspring show a larger variability in comparison with the original clone. This aspect remains a major limiting factor in the application of CRISPR/Cas9 via stable transformation for improving woody fruit crop species. The most common alternative now proposed is the transient cell transformation by the insertion of the CRISPR/Cas9 Ribonucleoproteins (RNPs) complex in protoplast cells (Malnoy et al., 2016). In this case, no stable genetic modification occurs, while the inserted protein complex induces the mutation, the CRISPR/Cas9 RNPs are quickly cleared from the cell via protein degradation pathways resulting in a GM free gene-edited plant. This could be considered the most appropriate approach for applying CRISPR/Cas9 to induce point mutations, or for gene insertion/knockdown in woody fruit species. However, a major limitation is the inability to regenerate plants from modified protoplasts of many important woody fruit species (Mezzetti et al., 2001), indeed few examples exist of optimal regeneration protocols for woody fruit species protoplasts (Patat-Ochatt et al., 1988; Vardi et al., 1990).

## Conclusions

Biotechnological techniques have undergone rapid development adding novel and valuable tools for plant breeders. These techniques make it possible to create desirable crop cultivars in fast and more efficient ways to meet the demand for improved crops to support sustainable agricultural productivity and in order to cater for the ever-increasing world population.

Although the new biotechnological techniques have one common goal i.e., precise, fast, and efficient crop improvement, individually they are different in approach and characteristics from each other. Some of these techniques, such as RNAi and trans-grafting, can be combined to achieve the desired results. Commercial applications of genetically modified fruit trees are so far limited. The only fruit plants available on the market are the “Rainbow” virus resistant papaya since September 1997, when all the necessary procedures for approval had been completed successfully (Gonsalves, 2004), and the arctic apple approved by the US Department of Agriculture (USDA) in February 2015, making it the first genetically modified apple resistant to browning (Waltz, 2015). The virus resistant Honey Sweet plum cultivar attained approval for commercialization in USA but has not reached the market yet (Scorza et al., 2013). The limited application of GM technology in fruit trees can be explained by (1) the difficulties in developing efficient regeneration and transformation protocols for many cultivars of the different species as many fruit tree species are recalcitrant, (2) the regulatory requirements. These reasons lead to the limited commercial exploitation of GM fruit trees by the fruit industry hence limited investment in fruit tree biotechnologies by plant breeders and the biotech industry. In this connection therefore, it is mainly public research institutions, with limited budgets, that are developing biotechnology research on these crops.

The NBTs such as cisgenesis and intragenesis could raise less biosafety concerns and should be considered more similar to conventional breeding techniques; RNAi introduces no new proteins in the plant, which means no novel allergenicity issues and that a lightened risk assessment process should be required. Furthermore, gene editing techniques, especially CRISPR/Cas9 combined with RNPs delivered directly to the protoplast, are more precise and targeted techniques and less likely to create unintended off-target mutations as RNPs are quickly cleared from the cell via protein degradation pathways resulting in a modified plant free from any foreign materials from the CRISPR/Cas9 RNPs complex.

## Author contributions

CL designed and contributed to the writing of the main body of article; SS contributed by writing specific parts of the article and revising it critically; JS contributed to the development of specific parts of the article and to its critical revision; BM conceived and contributed toward the writing of specific parts of the article and in its critical revision.

### Conflict of interest statement

The authors declare that the research was conducted in the absence of any commercial or financial relationships that could be construed as a potential conflict of interest. The reviewer UM and handling Editor declared their shared affiliation, and the handling Editor states that the process met the standards of a fair and objective review.

## References

[B1] Abel P. P. Nelson R. S. De B. Hoffmann N. Rogers S. G. Fraley R. T.. (1986). Delay of disease development in transgenic plants that express the tobacco mosaic virus coat protein gene. Science 232, 738–743. 10.1126/science.3457472 3457472

[B2] Almeida R. Allshire R. C. (2005). RNA silencing and genome Regulation. Trends Cell Biol. 15, 251–258. 10.1016/j.tcb.2005.03.006 15866029

[B3] Aloni B. Cohen R. Karni L. Aktas H. Edelstein M. (2010). Hormonal signaling in rootstock-scion interactions. Sci. Hortic. 127, 119–126. 10.1016/j.scienta.2010.09.003

[B4] Altpeter F. Baisakh N. Beachy R. Bock R. Capell T. Christou P. (2005). Particle bombardment and the genetic enhancement of crops: myths and realities. Mol. Breed. 15, 305–327. 10.1007/s11032-004-8001-y

[B5] An C. Orbović V. Mou Z. (2013). An efficient intragenic vector for generating intragenic and cisgenic plants in citrus. Am. J. Plant Sci. 4, 2131–2137. 10.4236/ajps.2013.411265

[B6] Anders C. Niewoehner O. Duerst A. Jinek M. (2014). Structural basis of PAM-dependent target DNA recognition by the Cas9 endonuclease. Nature 513, 569–573. 10.1038/nature13579 25079318 PMC4176945

[B7] Atkinson R. G. Sutherland P. W. Johnston S. L. Gunaseelan K. Hallett I. C. Mitra D.. (2012). Down-regulation of *POLYGALACTURONASE*1 alters firmness, tensile strength and water loss in apple (*Malus x domestica*) fruit. BMC Plant Biol. 12:129. 10.1186/1471-2229-12-129 22856470 PMC3509026

[B8] Barrangou R. (2015). The roles of CRISPR–Cas systems in adaptive immunity and beyond. Curr. Opin. Immunol. 32, 36–41. 10.1016/j.coi.2014.12.008 25574773

[B9] Bartel D. P. (2004). MicroRNAs: genomics, biogenesis, mechanism and function. Cell 116, 281–297. 10.1016/S0092-8674(04)00045-5 14744438

[B10] Baulcombe D. C. (1996). Mechanisms of pathogen-derived resistance to viruses in transgenic plants. Plant Cell 8, 1833–1844. 10.1105/tpc.8.10.1833 12239365 PMC161318

[B11] Baulcombe D. C. (2004). RNA silencing in plant. Nature 431, 356–363. 10.1038/nature02874 15372043

[B12] Baum J. A. Bogaert T. Clinton W. Heck G. R. Feldmann P. Ilagan O.. (2007). Control of coleopteran insect pests through RNA interference. Nat. Biotechnol. 25, 1322–1326. 10.1038/nbt1359 17982443

[B13] Bhaya D. Davison M. Barrangou R. (2011). CRISPR-Cas systems in bacteria and archaea: versatile small RNAs for adaptive defense and regulation. Annu. Rev. Genet. 45, 273–297. 10.1146/annurev-genet-110410-132430 22060043

[B14] Bhullar N. K. Gruissem W. (2013). Nutritional enhancement of rice for human health: the contribution of biotechnology. Biotechnol. Adv. 31, 50–57. 10.1016/j.biotechadv.2012.02.001 22343216

[B15] Billmyre R. B. Calo S. Feretzaki M. Wang X. Heitman J. (2013). RNAi function, diversity, and loss in the fungal kingdom. Chromosome Res. 21, 561–572. 10.1007/s10577-013-9388-2 24173579 PMC3874831

[B16] Boch J. Scholze H. Schornack S. Landgraf A. Hahn S. Kay S.. (2009). Breaking the code of DNA binding specificity of TAL-type III effectors. Science 326, 1509–1512. 10.1126/science.1178811 19933107

[B17] Borges F. Martienssen R. A. (2015). The expanding world of small RNAs in plants. Nat. Rev. Mol. Cell Biol. 16, 727–741. 10.1038/nrm4085 26530390 PMC4948178

[B18] Bortesi L. Fischer R. (2015). The CRISPR/Cas9 system for plant genome editing and beyond. Biotechnol. Adv. 33, 41–52. 10.1016/j.biotechadv.2014.12.006 25536441

[B19] Brazelton V. A. Jr. Zarecor S. Wright D. A. Wang Y. Liu J. Chen K.. (2016). A quick guide to CRISPR sgRNA design tools. GM Crops Food 6, 266–276. 10.1080/21645698.2015.1137690 26745836 PMC5033207

[B20] Breyer D. Herman P. Brandenburger A. Gheysen G. Remaut E. Soumillion P. (2009). Commentary: genetic modification through oligonucleotide-mediated mutagenesis. A GMO regulatory challenge? Environ. Biosaf. Res. 8, 57–64. 10.1051/ebr/2009007 19833073

[B21] Brodersen P. Voinnet O. (2006). The diversity of RNA silencing pathways in plants. Trends Genet. 22, 268–280. 10.1016/j.tig.2006.03.003 16567016

[B22] Brodersen P. Sakvarelidze-Achard L. Bruun-Rasmussen M. Dunoyer P. Yamamoto Y. Y. Sieburth L.. (2008). Widespread translational inhibition by plant miRNAs and siRNAs. Science 320, 1185–1190. 10.1126/science.1159151 18483398

[B23] Brosnan C. A. Voinnet O. (2011). Cell-to-cell and long-distance siRNA movement in plants: mechanisms and biological implications. Curr. Opin. Plant Biol. 14, 580–587. 10.1016/j.pbi.2011.07.011 21862389

[B24] Cambra M. Capote N. Myrta A. Llácer G. (2006). Plum pox virus and estimated costs associated to sharka disease. Bull. OEPP/EPPO Bull. 36, 202–204. 10.1111/j.1365-2338.2006.01027.x

[B25] Campbell T. N. Choy F. Y. (2005). RNA interference: past, present and future. Curr. Issues Mol. Biol. 7, 1–6. 15580776

[B26] Carbonell A. Carrington J. C. (2015). Antiviral roles of plant ARGONAUTES. Curr. Opin. Plant Biol. 27, 111–117. 10.1016/j.pbi.2015.06.013 26190744 PMC4618181

[B27] Carroll D. (2011). Genome engineering with zinc-finger nucleases. Genetics 188, 773–782. 10.1534/genetics.111.131433 21828278 PMC3176093

[B28] Carthew R. W. Sontheimer E. J. (2009). Origins and Mechanisms of miRNAs and siRNAs. Cell 136, 642–655. 10.1016/j.cell.2009.01.035 19239886 PMC2675692

[B29] Cervera M. Juarez J. Navarro A. Pina J. A. Duran-Vila N. Navarro L. (1998). Genetic transformation and regeneration of mature tissues of woody fruit plants bypassing the juvenile stage. Transgenic Res. 7, 51–59. 10.1023/A:1008855922283

[B30] Cheng W. Song X. S. Li H. P. Cao L. H. Sun K. Qiu X. L.. (2015). Host induced gene silencing of an essential chitin synthase gene confers durable resistance to Fusarium head blight and seedling blight in wheat. Plant Biotechnol. J. 13, 1335–1345. 10.1111/pbi.12352 25735638

[B31] Chilton M. D. Drummond M. H. Merio D. J. Sciaky D. Montoya A. L. Gordon M. P.. (1977). Stable incorporation of plasmid DNA into higher plant cells: the molecular basis of crown gall tumorigenesis. Cell 11, 263–271. 10.1016/0092-8674(77)90043-5 890735

[B32] Collinge D. B. Jørgensen H. J. Lund O. S. Lyngkjær M. F. (2010). Engineering pathogen resistance in crop plants: current trends and future prospects. Annu. Rev. Phytopathol. 48, 269–291. 10.1146/annurev-phyto-073009-114430 20687833

[B33] Cong L. Ran F. A. Cox D. Lin S. Barretto R. Habib N.. (2013). Multiplex genome engineering using CRISPR/Cas systems. Science 339, 819–823. 10.1126/science.1231143 23287718 PMC3795411

[B34] Conner A. J. Barrell P. J. Baldwin S. J. Lokerse A. S. Cooper P. A. Erasmuson A. K. (2007). Intragenic vectors for gene transfer without foreign DNA. Euphytica 154, 341–353. 10.1007/s10681-006-9316-z

[B35] Curtin S. J. Voytas D. F. Stupar R. M. (2012). Genome engineering of crops with designer nucleases. Plant Genome 5, 42–50. 10.3835/plantgenome2012.06.0008

[B36] Curtin S. J. Zhang F. Sander J. D. Haun W. J. Starker C. Baltes N. J.. (2011). Targeted mutagenesis of duplicated genes in soybean with zinc-finger nucleases. Plant Physiol. 156, 466–473. 10.1104/pp.111.172981 21464476 PMC3177250

[B37] Datta A. (2013). Genetic engineering for improving quality and productivity of crops. Agric. Food Security 2:15 10.1186/2048-7010-2-15

[B38] De Alba A. E. M. Elvira-Matelot E. Vaucheret H. (2013). Gene silencing in plants: a diversity of pathways. Biochim. Biophys. Acta Gene Regul. Mech. 1829, 1300–1308. 10.1016/j.bbagrm.2013.10.005 24185199

[B39] Deng D. Yan C. Pan X. Mahfouz M. Wang J. Zhu J. K.. (2012). Structural basis for sequence-specific recognition of DNA by TAL effectors. Science 335, 720–723. 10.1126/science.1215670 22223738 PMC3586824

[B40] Dhekney S. A. Li Z. T. Gray D. J. (2011). Grapevines engineered to express cisgenic *Vitis vinifera* thaumatin-like protein exhibit fungal disease resistance *in vitro* cell. Dev. Biol. Plant 47, 458–466. 10.1007/s11627-011-9358-3

[B41] Ding S. W. (2010). RNA-based antiviral immunity. Nat. Rev. Immunol. 10, 632–644. 10.1038/nri2824 20706278

[B42] Ding S. W. Voinnet O. (2007). Antiviral immunity directed by small RNAs. Cell 130, 413–426. 10.1016/j.cell.2007.07.039 17693253 PMC2703654

[B43] Dong C. Beetham P. Vincent K. Sharp P. (2006). Oligonucleotide-directed gene repair in wheat using a transient plasmid gene repair assay system. Plant Cell Rep. 25, 457–465. 10.1007/s00299-005-0098-x 16404599

[B44] Dunoyer P. Himber C. Voinnet O. (2005). DICER-LIKE 4 is required for RNA interference and produces the 21-nucleotide small interfering RNA component of the plant cell-to-cell silencing signal. Nat. Genet. 37, 1356–1360. 10.1038/ng1675 16273107

[B45] Dutt M. Li Z. T. Kelley K. T. Dhekney S. A. Van Aman M. Tattersall J. (2007). Transgenic rootstock protein transmission in grapevines. Acta Hortic. 738, 749–754. 10.17660/ActaHortic.2007.738.99

[B46] Elomaa P. Uimari A. Mehto M. Albert V. A. Laitinen R. A. Teeri T. H. (2003). Activation of anthocyanin biosynthesis in Gerbera hybrida (Asteraceae) suggests conserved protein-protein and protein-promoter interactions between the anciently diverged monocots and eudicots. Plant Physiol. 133, 1831–1842. 10.1104/pp.103.026039 14605235 PMC300736

[B47] English J. J. Mueller E. Baulcombe D. C. (1996). Suppression of virus accumulation in transgenic plants exhibiting silencing of nuclear genes. Plant Cell 8, 179–188. 10.1105/tpc.8.2.179 12239381 PMC161090

[B48] Escobar M. A. Civerolo E. L. Summerfelt K. R. Dandekar A. M. (2001). RNAi-mediated oncogene silencing confers resistance to crown gall tumorigenesis. Proc. Nat. Acad. Sci. U.S.A. 98, 13437–13442. 10.1073/pnas.241276898 11687652 PMC60889

[B49] European Food Safety Authority (EFSA) (2012). Scientific opinion addressing the safety assessment of plants developed through cisgenesis and intragenesis. EFSA J. 10:2561 10.2903/j.efsa.2012.2561 PMC9583739 36274982

[B50] Febres V. J. Lee R. F. Moore G. A. (2008). Transgenic resistance to Citrus tristeza virus in grapefruit. Plant Cell Rep. 27, 93–104. 10.1007/s00299-007-0445-1 17882423

[B51] Fire A. Xu S. Q. Montgomery M. K. Kostas S. A. Driver S. E. Mello C. C. (1998). Potent and specific genetic interference by double-stranded RNA in *Caenorhabditis elegans*. Nature 391, 806–811. 10.1038/35888 9486653

[B52] Freiman A. Shlizerman L. Golobovitch S. Yablovitz Z. Korchinsky R. Cohen Y.. (2012). Development of a transgenic early flowering pear (*Pyrus communis* L.) genotype by RNAi silencing of *PcTFL1-1* and *PcTFL1-2*. Planta 235, 1239–1251. 10.1007/s00425-011-1571-0 22203321

[B53] Frizzi A. Huang S. (2010). Tapping RNA silencing pathways for plant biotechnology. Plant Biotechnol. J. 8, 655–677. 10.1111/j.1467-7652.2010.00505.x 20331529

[B54] Funke T. Han H. Healy-Fried M. L. Fischer M. Schönbrunn E. (2006). Molecular basis for the herbicide resistance of Roundup Ready crops. Proc. Natl. Acad. Sci. U.S.A. 103, 13010–13015. 10.1073/pnas.0603638103 16916934 PMC1559744

[B55] Gaj T. Gersbach C. A. Barbas C. F. (2013). ZFN, TALEN, and CRISPR/Cas-based methods for genome engineering. Trends Biotechnol. 31, 397–405. 10.1016/j.tibtech.2013.04.004 23664777 PMC3694601

[B56] Gelvin S. B. (2003). Agrobacterium-mediated plant transformation: the biology behind the “gene-jockeying” tool. Microbiol. Mol. Biol. Rev. 67, 16–37. 10.1128/MMBR.67.1.16-37.2003 12626681 PMC150518

[B57] Gessler C. Vanblaere T. Parravicini G. Broggini G. A. L. (2014). Cisgenic ‘Gala’ containing the scab resistance gene from *Malus floribunda* 821 and the fire blight resistance genes from *M. ‘Evereste’*. Acta Horticult. 1048, 43–50. 10.17660/ActaHortic.2014.1048.4

[B58] Giuliano G. (2017). Provitamin A biofortification of crop plants: a gold rush with many miners. Curr. Opin. Biotechnol. 44, 169–180. 10.1016/j.copbio.2017.02.001 28254681

[B59] Gonsalves D. (1998). Control of papaya ringspot virus in papaya: a case study. Annu. Rev. Phytopathol. 36, 415–437. 10.1146/annurev.phyto.36.1.415 15012507

[B60] Gonsalves D. (2004). Transgenic papaya in Hawaii and beyond. AgBioForum 7, 36–40.

[B61] Gonsalves D. (2006). Transgenic papaya: development, release, impact and challenges. Adv. Virus Res. 67, 317–354. 10.1016/S0065-3527(06)67009-7 17027684

[B62] Gottula J. Fuchs M. (2009). Toward a quarter century of pathogen derived resistance and practical approaches to plant virus disease control. Adv. Virus Res. 75, 161–183. 10.1016/S0065-3527(09)07505-8 20109666

[B63] Grens K. (2017). RNA Interference Between Kingdoms. Available online at: http://www.the-scientist.com/?articles.view/articleNo/48073/title/RNAInterferenceBetweenKingdoms/&_hsenc=p2ANqtz-_gT8pO7h21CVKZggjSAJSIGfBMHMB7hsrba2Ggg82zZCKW6skKkR8qLPrY_M7AxCl0394O9EFjs14yUYwSAKKv1zQ&_hsmi=41791015/

[B64] Hannon G. J. (2002). RNA interference. Nature 418, 244–251. 10.1038/418244a 12110901

[B65] Haroldsen V. M. Chi-Ham C. L. Bennett A. B. (2012a). Transgene mobilization and regulatory uncertainty for non-GE fruit products of transgenic rootstocks. J. Biotechnol. 161, 349–353. 10.1016/j.jbiotec.2012.06.017 22749907

[B66] Haroldsen V. M. Szczerba M. W. Aktas H. Lopez-baltazar J. Odias M. J. Chi-ham C. L.. (2012b). Mobility of transgenic nucleic acids and proteins within grafted rootstocks for agricultural improvement. Front. Plant Sci. 3:39. 10.3389/fpls.2012.00039 22645583 PMC3355758

[B67] Hartung F. Schiemann J. (2014). Precise plant breeding using new genome editing techniques: opportunities, safety and regulation in the EU. Plant J. 78, 742–752. 10.1111/tpj.12413 24330272

[B68] Hily J. M. Ravelonandro M. Damsteegt V. Bassett C. Petri C. Liu Z. (2007). Plum pox virus coat protein gene Intron-hairpin-RNA (ihpRNA) constructs provide resistance to plum pox virus in Nicotiana benthamiana and *Prunus domestica*. J. Am. Soc. Hortic. Sci. 132, 850–858.

[B69] Horvath P. Barrangou R. (2010). CRISPR/Cas, the immune system of Bacteria and Archaea. Science 327, 167. 10.1126/science.1179555 20056882

[B70] Hu Y. Zhang J. L. Jia H. G. Sosso D. Li T. Frommer W. B. Yang B.. (2014). Lateral organ boundaries 1 is a disease susceptibility gene for citrus bacterial canker disease. Proc. Natl. Acad. Sci. U.S.A. 111, E521–E529. 10.1073/pnas.1313271111 24474801 PMC3910620

[B71] Iida S. Terada R. (2005). Modification of endogenous natural genes by gene targeting in rice and other higher plants. Plant Mol. Biol. 59, 205–219. 10.1007/s11103-005-2162-x 16217613

[B72] Ipsaro J. J. Joshua-Tor L. (2015). From guide to target: molecular insights into eukaryotic RNA-interference machinery. Nat. Struct. Mol. Biol. 22, 20–28. 10.1038/nsmb.2931 25565029 PMC4450863

[B73] Jacobsen E. Schouten H. J. (2007). Cisgenesis strongly improves introgression breeding and induced translocation breeding of plants. Trends Biotechnol. 25, 219–223. 10.1016/j.tibtech.2007.03.008 17383037

[B74] Jansen R. Embden J. D. Gaastra W. Schouls L. M. (2002). Identification of genes that are associated with DNA repeats in prokaryotes. Mol. Microbiol. 43, 1565–1575. 10.1046/j.1365-2958.2002.02839.x 11952905

[B75] Jia H. Wang N. (2014). Targeted genome editing of sweet orange using Cas9/sgRNA. PLoS ONE 9:e93806. 10.1371/journal.pone.0093806 24710347 PMC3977896

[B76] Jiang W. Bikard D. Cox D. Zhang F. Marraffini L. A. (2013). RNA-guided editing of bacterial genomes using CRISPR-Cas systems. Nat. Biotechnol. 31, 233–239. 10.1038/nbt.2508 23360965 PMC3748948

[B77] Jinek M. Chylinski K. Fonfara I. Hauer M. Doudna J. A. Charpentier E. (2012). A programmable dual-RNA-guided DNA endonuclease in adaptive bacterial immunity. Science 337, 816–821. 10.1126/science.1225829 22745249 PMC6286148

[B78] Jonas S. Izaurralde E. (2015). Towards a molecular understanding of microRNA-mediated gene silencing. Nat. Rev. Genet. 16, 421–433. 10.1038/nrg3965 26077373

[B79] Jones H. D. (2015). Regulatory uncertainty over genome editing. Nat. Plants 1, 14011. 10.1038/nplants.2014.11 27246057

[B80] Joshi S. G. Schaart J. G. Groenwold R. Jacobsen E. Schouten H. J. Krens F. A. (2011). Functional analysis and expression profiling of *HcrVf1* and *HcrVf2* for development of scab resistant cisgenic and intragenic apples. Plant Mol. Biol. 75, 579–591. 10.1007/s11103-011-9749-1 21293908 PMC3057008

[B81] Kandel R. Bergey D. R. Dutt M. Sitther V. Li Z. T. Gray D. J. (2016). Evaluation of a grapevine-derived reporter gene system for precision breeding of *Vitis*. Plant Cell Tiss. Organ Cult. 124, 599–609. 10.1007/s11240-015-0918-9

[B82] Ketting R. F. (2011). The many faces of RNAi. Dev. Cell. 20, 148–161. 10.1016/j.devcel.2011.01.012 21316584

[B83] Khalid A. Zhang Q. Yasir M. Li F. (2017). Small RNA based genetic engineering for plant viral resistance: application in crop protection. Front. Microbiol. 8:43. 10.3389/fmicb.2017.00043 28167936 PMC5253543

[B84] Klocko A. L. Borejsza-Wysocka E. Brunner A. M. Shevchenko O. Aldwinckle H. Strauss S. H. (2016). Transgenic suppression of *AGAMOUS* genes in apple reduces fertility and increases floral attractiveness. PLoS ONE 11:e0159421. 10.1371/journal.pone.0159421 27500731 PMC4976969

[B85] Knip M. Constantin M. E. Thordal-Christensen H. (2014). Trans-kingdom cross-talk: small RNAs on the move. PLoS Genet. 10:e1004602. 10.1371/journal.pgen.1004602 25188222 PMC4154666

[B86] Kost T. D. Gessler C. Jänsch M. Flachowsky H. Patocchi A. Broggini G. A. L. (2015). Development of the first cisgenic apple with increased resistance to fire blight. PLoS ONE 10:e0143980. 10.1371/journal.pone.0143980 26624292 PMC4666654

[B87] Krens F. A. Schaart J. G. Van der Burgh A. M. Tinnenbroek-Capel I. E. Groenwold M. R. Kodde L. P.. (2015). Cisgenic apple trees; development, characterization, and performance. Front. Plant Sci. 6:286. 10.3389/fpls.2015.00286 25964793 PMC4410516

[B88] Lang A. Chailakhyan M. K. Frolova I. A. (1977). Promotion and inhibition of flower formation in a day neutral plant in grafts with a short-day plant and a long-day plant. Proc. Natl. Acad. Sci. U.S.A. 74, 2412–2416. 10.1073/pnas.74.6.2412 16592404 PMC432182

[B89] Lemgo G. Sabbadini S. Pandolfini T. Mezzetti B. (2013). Biosafety considerations of RNAi-mediated virus resistance in fruit-tree cultivars and in rootstock. Transgenic Res. 22, 1073–1088. 10.1007/s11248-013-9728-1 23857556

[B90] Lombardo L. Coppola G. Zelasco S. (2016). New technologies for insect-resistant and herbicide-tolerant plants. Trends Biotechnol. 34, 49–57. 10.1016/j.tibtech.2015.10.006 26620971

[B91] Lucas W. J. Lee J.-Y. (2004). Plasmodesmata as a supracellular control network in plants. Nat. Rev. Mol. Cell Biol. 5, 712–726. 10.1038/nrm1470 15340379

[B92] Lusser M. Davies H. V. (2013). Comparative regulatory approaches for groups of new plant breeding techniques. Nat. Biotechnol. 30, 437–446. 10.1016/j.nbt.2013.02.004 23474021

[B93] Lusser M. Parisi C. Plan D. Rodríguez-Cerezo E. (2011). New Plant Breeding Techniques. State of-the-Art And Prospects for Commercial Development. JRC Scientific and Technical Reports/EUR 24760 EN. European Commission.

[B94] Lusser M. Parisi C. Plan D. Rodríguez-Cerezo E. (2012). Deployment of new biotechnologies in plant breeding. Nat. Biotechnol. 30, 231–239. 10.1038/nbt.2142 22398616 PMC7097357

[B95] Mallory A. C. Mlotshwa S. Bowman L. H. Vance V. B. (2003). The capacity of transgenic tobacco to send a systemic RNA silencing signal depends on the nature of the inducing transgene locus. Plant J. 35, 82–92. 10.1046/j.1365-313X.2003.01785.x 12834404

[B96] Malnoy M. Viola R. Jung M.-H. Koo O.-J. Kim S. Kim J.-S.. (2016). DNA-free genetically edited grapevine and apple protoplast using CRISPR/Cas9 ribonucleoproteins. Front. Plant Sci. 7:1904. 10.3389/fpls.2016.01904 28066464 PMC5170842

[B97] Matzke M. A. Mosher R. A. (2014). RNA-directed DNA methylation: an epigenetic pathway of increasing complexity. Nat. Rev. Genet. 15, 394–408. 10.1038/nrg3683 24805120

[B98] Matzke M. A. Matzke A. J. Pruss G. J. Vance V. B. (2001). RNA-based silencing strategies in plants. Curr. Opin. Genet. Dev. 11, 221–227. 10.1016/S0959-437X(00)00183-0 11250148

[B99] McGarry R. C. Kragler F. (2013). Phloem-mobile signals affecting flowers: applications for crop breeding. Trends Plant Sci. 18, 198–206. 10.1016/j.tplants.2013.01.004 23395308

[B100] Meister G. Tuschl T. (2004). Mechanisms of gene silencing by double-stranded RNA. Nature 431, 343–349. 10.1038/nature02873 15372041

[B101] Melnyk C. W. Meyerowitz E. M. (2015). Plant grafting. Curr. Biol. 25, R183–R188. 10.1016/j.cub.2015.01.029 25734263

[B102] Melnyk C. W. Molnar A. Baulcombe D. C. (2011). Intercellular and systemic movement of RNA silencing signals. EMBO J. 30, 3553–3563. 10.1038/emboj.2011.274 21878996 PMC3181474

[B103] Metzlaff M. O'dell M. Cluster P. D. Flavell R. B. (1997). RNA-mediated RNA degradation and chalcone synthase A silencing in petunia. Cell 88, 845–854. 9118227 10.1016/s0092-8674(00)81930-3

[B104] Mezzetti B. Landi L. Phan B. H. Taruschio L. Lim Y. K. (2001). PEG-mediated fusion of *Rubus idaeus* (raspberry) and *R. fructicosus* (blackberry) and protoplast, selection and characterization of callus lines. Plant Biosyst. 135, 63–70. 10.1080/11263500112331350660

[B105] Mezzetti B. Pandolfini T. Navacchi O. Landi L. (2002). Genetic transformation of *Vitis vinifera* via organogenesis. BMC Biotechnol. 2:18. 10.1186/1472-6750-2-18 12354328 PMC130035

[B106] Miller J. C. Holmes M. C. Wang J. Guschin D. Y. Lee Y. L. Rupniewski I.. (2007). An improved zinc-finger nuclease architecture for highly specific genome editing. Nat. Biotechnol. 25, 778–785. 10.1038/nbt1319 17603475

[B107] Mittler R. Blumwald E. (2010). Genetic engineering for modern agriculture: challenges and perspectives. Annu. Rev. Plant Biol. 61, 443–462. 10.1146/annurev-arplant-042809-112116 20192746

[B108] Molnar A. Melnyk C. W. Bassett A. Hardcastle T. J. Dunn R. Baulcombe D. C. (2010). Small silencing RNAs in plants are mobile and direct epigenetic modification in recipient cells. Science 328, 872–875. 10.1126/science.1187959 20413459

[B109] Molnar A. Melnyk C. Baulcombe D. C. (2011). Silencing signals in plants: a long journey for small RNAs. Genome Biol. 12:215. 10.1186/gb-2010-11-12-219 21235831 PMC3091295

[B110] Moscou M. J. Bogdanove A. J. (2009). A simple cipher governs DNA recognition by TAL effectors. Science 326:1501. 10.1126/science.1178817 19933106

[B111] Mudge K. Janick J. Scofield S. Goldschmidt E. E. (2009). A history of grafting. Hortic. Rev. 35, 437–493. 10.1002/9780470593776.ch9

[B112] Nagamangala K. C. Sargent D. J. Velasco R. Maffei M. E. Malnoy M. (2015). Looking forward to genetically edited fruit crops. Trends Biotechnol. 33, 62–64. 10.1016/j.tibtech.2014.07.003 25129425

[B113] Nakajima I. Ban Y. Azuma A. Onoue N. Moriguchi T. Yamamoto T.. (2017). CRISPR/Cas9-mediated targeted mutagenesis in grape. PLoS ONE 12:e0177966. 10.1371/journal.pone.0177966 28542349 PMC5436839

[B114] Nakayashiki H. (2005). RNA silencing in fungi: mechanisms and applications. FEBS Lett. 579, 5950–5970. 10.1016/j.febslet.2005.08.016 16137680

[B115] Napoli C. Lemieux C. Jorgensen R. (1990). Introduction of a chimeric chalcone synthase gene into petunia results in reversible co-suppression of homologous genes in trans. Plant Cell 2, 279–289. 10.1105/tpc.2.4.279 12354959 PMC159885

[B116] Navarro L. Dunoyer P. Jay F. Arnold B. Dharmasiri N. Estelle M.. (2006). A plant miRNA contributes to antibacterial resistance by repressing auxin signaling. Science 312, 436–439. 10.1126/science.1126088 16627744

[B117] Nazim U. M. Kim J. Y. (2013). Intercellular and systemic spread of RNA and RNAi in plants. Wiley Interdiscip. Rev. RNA. 4, 279–293. 10.1002/wrna.1160 23536229

[B118] Nishimasu H. Ran F. A. Hsu P. D. Konermann S. Shehata S. I. Dohmae N.. (2014). Crystal structure of Cas9 in complex with guide RNA and target DNA. Cell 156, 935–949. 10.1016/j.cell.2014.02.001 24529477 PMC4139937

[B119] Nishitani C. Hirai S. K. Masato W. Kazuma O. Keishi O. Toshiya Y.. (2016). Efficient genome editing in apple using a CRISPR/Cas9 system. Sci. Rep. 6:31481. 10.1038/srep31481 27530958 PMC4987624

[B120] Omid A. Keilin T. Glass A. Leshkowitz D. Wolf S. (2007). Characterization of phloem-sap transcription profile in melon plants. J. Exp. Bot. 58, 3645–3656. 10.1093/jxb/erm214 17928373

[B121] Osakabe Y. Osakabe K. (2015). Genome editing with engineered nucleases in plants. Plant Cell Physiol. 5, 389–400. 10.1093/pcp/pcu170 25416289

[B122] Pabo C. O. Peisach E. Grant R. A. (2001). Design and selection of novel Cys2His2 zinc finger proteins. Annu. Rev. Biochem. 70, 313–340. 10.1146/annurev.biochem.70.1.313 11395410

[B123] Paine J. Shipton C. Chaggar S. Howells R. Kennedy M. Vernon G.. (2005). Improving the nutritional value of Golden Rice through increased pro-vitamin A content. Nat. Biotechnol. 23, 482–487. 10.1038/nbt1082 15793573

[B124] Palauqui J. C. Elmayan T. Pollien J. M. Vaucheret H. (1997). Systemic acquired silencing: transgene-specific post-transcriptional silencing is transmitted by grafting from silenced stocks to non-silenced scions. EMBO J. 16, 4738–4745. 10.1093/emboj/16.15.4738 9303318 PMC1170100

[B125] Parent J. S. Vaucheret H. (2012). The origin and effect of small RNA signaling in plants. Front. Plant Sci. 3:179. 10.3389/fpls.2012.00179 22908024 PMC3414853

[B126] Parisi C. Tillie P. Rodríguez-Cerezo E. (2016). The global pipeline of GM crops out to 2020. Nat. Biotechnol. 34, 32–36. 10.1038/nbt.3449 26744975

[B127] Patat-Ochatt E. M. Ochatt S. J. Power J. B. (1988). Plant regeneration from protoplasts of apple rootstocks and scion varieties (*Malus* x *domestica* Borkh.). J. Plant Physiol. 133, 460–465. 10.1016/S0176-1617(88)80037-3

[B128] Peer R. Rivlin G. Golobovitch S. Lapidot M. Gal-On A. Vainstein A.. (2015). Targeted mutagenesis using zinc-finger nucleases in perennial fruit trees. Planta 241, 941–951. 10.1007/s00425-014-2224-x 25528147

[B129] Peil A. Garcia-Libreros T. Richter K. Trognitz F. C. Trognitz B. Hanke M. V. (2007). Strong evidence for a fire blight resistance gene of Malus robusta located on linkage group 3. Plant Breed. 126, 470–475. 10.1111/j.1439-0523.2007.01408.x

[B130] Peng A. Chen S. Lei T. Xu L. He Y. Wu L. (2017). Engineering canker-resistant plants through CRISPR/Cas9-targeted editing of the susceptibility gene *CsLOB*1 promoter in citrus. Plant Biotech. J. [Epub ahead of print]. 10.1111/pbi.12733 PMC5698050 28371200

[B131] Pérez-Jiménez M. Carrillo-Navarro A. Cos-Terrer J. (2012). Regeneration of peach (*Prunus persica* L. Batsch) cultivars and *Prunus persica*× *Prunus dulcis* rootstocks via organogenesis. Plant Cell Tissue Organ Cult. 108, 55–62. 10.1007/s11240-011-0011-y

[B132] Pérez-Massot E. Banakar R. Gómez-Galera S. Zorrilla-López U. Sanahuja G. Arjó G.. (2013). The contribution of transgenic plants to better health through improved nutrition: opportunities and constraints. Genes Nutr. 8, 29–41. 10.1007/s12263-012-0315-5 22926437 PMC3534993

[B133] Pessina S. Angeli D. Martens S. Visser R. Bai Y. Salamini F.. (2016). The knock-down of the expression of *MdMLO19* reduces susceptibility to powdery mildew (*Podosphaera leucotricha*) in apple (*Malus domestica*). Plant Biotechnol. J. 14, 2033–2044. 10.1111/pbi.12562 26997489 PMC5043462

[B134] Petolino J. F. (2015). Genome editing in plants via designed zinc finger nucleases. In Vitro Cell. Dev. Biol. 51:1. 10.1007/s11627-015-9663-3 25774080 PMC4352198

[B135] Petri C. Burgos L. (2005). Transformation of fruit trees. Useful breeding tool or continued future prospect? Transgenic Res. 14, 15–26. 10.1007/s11248-004-2770-2 15865045

[B136] Podevin N. Devos Y. Davies H. V. Nielsen K. M. (2012). Transgenic or not? No simple answer! New biotechnology-based plant breeding techniques and the regulatory landscape. EMBO Rep. 13, 1057–1061. 10.1038/embor.2012.168 23154464 PMC3512411

[B137] Qaim M. Kouser S. (2013). Genetically modified crops and food security. PLoS ONE 8:e64879. 10.1371/journal.pone.0064879 23755155 PMC3674000

[B138] Qi Y. Hannon G. J. (2005). Uncovering RNAi mechanisms in plants: biochemistry enters the foray. FEBS Lett. 579, 5899–5903. 10.1016/j.febslet.2005.08.035 16154569

[B139] Rai M. K. Shekhawat N. S. (2014). Recent advances in genetic engineering for improvement of fruit crops. Plant Cell Tissue Organ Cult 116, 1–15. 10.1007/s11240-013-0389-9

[B140] Ren C. Liu X. Zhang Z. Wang Y. Duan W. Li S.. (2016). CRISPR/Cas9-mediated efficient targeted mutagenesis in Chardonnay (*Vitis vinifera* L.) Sci. Rep. 6:32289. 10.1038/srep32289 27576893 PMC5006071

[B141] Reyes C. A. Francesco A. D. Pe na E. J. Costa N. Plata M. I. Sendin L.. (2011). Resistance to *Citrus psorosis* virus in transgenic sweet orange plants is triggered by coat protein–RNA silencing. J. Biotechnol. 151, 151–158. 10.1016/j.jbiotec.2010.11.007 21084056

[B142] Romano N. Macino G. (1992). Quelling: transient inactivation of gene expression in *Neurospora crassa* by transformation with homologous sequences. Mol. Microbiol. 6, 3343–3353. 10.1111/j.1365-2958.1992.tb02202.x 1484489

[B143] Rommens C. M. (2004). All-native DNA transformation: a new approach to plant genetic engineering. Trends Plant Sci. 9, 457–464. 10.1016/j.tplants.2004.07.001 15337496

[B144] Rommens C. M. (2007). Intragenic crop improvement: combining the benefits of traditional breeding and genetic engineering. J. Agric. Food Chem. 55, 4281–4288. 10.1021/jf0706631 17488120

[B145] Ruiter R. Van Den Brande I. Stals E. Delauré S. Cornelissen M. D'halluin K. (2003). Spontaneous mutation frequency in plants obscures the effect of chimeraplasty. Plant Mol. Biol. 53, 715–729. 10.1023/B:PLAN.0000019111.96107.01 15010606

[B146] Sabbadini S. Pandolfini T. Girolomini L. Molesini B. Navacchi O. (2015). Peach (Prunus persica L.) in Agrobacterium Protocols, ed Wang K. (New York, NY: Springer), 205–215. 10.1007/978-1-4939-1658-0_17 25416260

[B147] Salame T. M. Ziv C. Hadar Y. Yarden O. (2011). RNAi as a potential tool for biotechnological applications in fungi. Appl. Microbiol. Biotechnol. 89, 501–512. 10.1007/s00253-010-2928-1 20953869

[B148] Sanford J. C. Johnston S. A. (1985). The concept of pathogen-derived resistance: deriving resistance genes from the parasite's own genome. J. Theor. Biol. 113, 395–405. 10.1016/S0022-5193(85)80234-4

[B149] Saporta R. San Pedro T. Gisbert C. (2017). Attempts at grapevine (*Vitis vinifera* L.) breeding through genetic transformation: the main limiting factors. VITIS J. Grape. Res. 55, 173–186. 10.5073/vitis.2016.55.173-186

[B150] Sauer N. J. Mozoruk J. Miller R. B. Warburg Z. J. Walker K. A. Beetham P. R.. (2016). Oligonucleotide-directed mutagenesis for precision gene editing. Plant Biotechnol. J. 14, 496–502. 10.1111/pbi.12496 26503400 PMC5057361

[B151] Schaart J. G. Visser R. G. F. (2009). Novel Plant Breeding Techniques - Consequences of New Genetic Modification-Based Techniques in Comparison to Conventional Plant Breeding. The Netherlands Commission on Genetic Modification. COGEM Research Report number 2009–02.

[B152] Schaart J. G. van de Wiel C. C. Lotz L. A. Smulders M. J. (2016). Opportunities for products of new plant breeding techniques. Trends Plant Sci. 21, 438–449. 10.1016/j.tplants.2015.11.006 26654659

[B153] Schouten H. J. Krens F. A. Jacobsen E. (2006a). Cisgenic plants are similar to traditionally bred plants: international regulations for genetically modified organisms should be altered to exempt cisgenesis. EMBO Rep. 7, 750–753. 10.1038/sj.embor.7400769 16880817 PMC1525145

[B154] Schouten H. J. Krens F. A. Jacobsen E. (2006b). Do cisgenic plants warrant less stringent oversight? Nat. Biotechnol. 24:753. 10.1038/nbt0706-753 16841052

[B155] Scorza R. Callahan A. Dardick C. Ravelonandro M. Polak J. Malinowski T. (2013). Genetic engineering of Plum pox virus resistance:‘HoneySweet’plum—from concept to product. Plant Cell Tissue Organ Cult. 115, 1–12. 10.1007/s11240-013-0339-6

[B156] Scorza R. Callahan A. Levy L. Damsteegt V. Webb K. Ravelonandro M. (2001). Post-transcriptional gene silencing in plum pox virus resistant transgenic European plum containing the plum pox poty virus coat protein gene. Transgenic Res. 10, 201–209. 10.1023/A:1016644823203 11437277

[B157] Scorza R. Ravelonandro M. Callahan A. M. Cordts J. M. Fuchs M. Dunez J. (1994). Transgenic plums (*Prunus domestica L*.) express the plum pox virus coat protein gene. Plant Cell Rep. 14, 18–22. 10.1007/BF00233291 24194220

[B158] Scott J. G. Michel K. Bartholomay L. C. Siegfried B. D. Hunter W. B. Smagghe G.. (2013). Towards the elements of successful insect RNAi. J. Insect Physiol. 59, 1212–1221. 10.1016/j.jinsphys.2013.08.014 24041495 PMC3870143

[B159] Simón-Mateo C. García J. A. (2011). Antiviral strategies in plants based on RNA silencing. BBA Gene Regul. Mech. 1809, 722–731. 10.1016/j.bbagrm.2011.05.011 21652000

[B160] Smith N. A. Singh S. P. Wang M. B. Stoutjesdijk P. A. Green A. G. Waterhouse P. M. (2000). Gene expression: total silencing by intron-spliced hairpin RNAs. Nature 407, 319–320. 10.1038/35030305 11014180

[B161] Smolka A. Li X. Y. Heikelt C. Welander M. Zhu L. H. (2010). Effects of transgenic rootstocks on growth and development of non-transgenic scion cultivars in apple. Transgenic Res. 19:933. 10.1007/s11248-010-9370-0 20135223

[B162] Song G. Q. Sink K. C. Walworth A. E. Cook M. A. Allison R. F. Lang G. A. (2013). Engineering cherry rootstocks with resistance to *Prunus necrotic ring spot virus* through RNAi-mediated silencing. Plant Biotechnol. J. 11, 702–708. 10.1111/pbi.12060 23521804

[B163] Sprink T. Eriksson D. Schiemann J. Hartung F. (2016). Regulatory hurdles for genome editing: process-vs. product-based approaches in different regulatory contexts. Plant Cell Rep. 35, 1493–1506. 10.1007/s00299-016-1990-2 27142995 PMC4903111

[B164] Stegemann S. Bock R. (2009). Exchange of genetic material between cells in plant tissue grafts. Science 324, 649–651. 10.1126/science.1170397 19407205

[B165] Stover E. Driggers R. Richardson M. L. Hall D. G. Duan Y. P. Lee R. F. (2014). Incidence and severity of asiatic citrus canker on diverse citrus and citrus-related germplasm in a FLORIDA field planting. HortScience 49, 4–9.

[B166] Townsend J. A. Wright D. A. Winfrey R. J. Fu F. Maeder M. L. Joung J. K.. (2009). High-frequency modification of plant genes using engineered zinc-finger nucleases. Nature 459, 442–445. 10.1038/nature07845 19404258 PMC2743854

[B167] Tzfira T. Citovsky V. (2006). Agrobacterium-mediated genetic transformation of plants: biology and biotechnology. Curr. Opin. Biotechnol. 17, 147–154. 10.1016/j.copbio.2006.01.009 16459071

[B168] Tzfira T. Weinthal D. Marton I. Zeevi V. Zuker A. Vainstein A. (2012). Genome modifications in plant cells by custom-made restriction enzymes. Plant Biotechnol. J. 10, 373–389. 10.1111/j.1467-7652.2011.00672.x 22469004

[B169] Urnov F. D. Rebar E. J. Holmes M. C. Zhang H. S. Gregory P. D. (2010). Genome editing with engineered zinc finger nucleases. Nat. Rev. Genet. 11, 636–646. 10.1038/nrg2842 20717154

[B170] Vanblaere T. Flachowsky H. Gessler C. Broggini G. A. (2014). Molecular characterization of cisgenic lines of apple ‘Gala’ carrying the *Rvi6* scab resistance gene. Plant Biotechnol. J. 12, 2–9. 10.1111/pbi.12110 23998808

[B171] Vanblaere T. Szankowski I. Schaart J. Schouten H. Flachowsky H. Broggini G. A. L.. (2011). The development of a cisgenic apple plant. J. Biotechnol. 154, 304–311. 10.1016/j.jbiotec.2011.05.013 21663775

[B172] Vardi A. Bleichman S. Aviv D. (1990). Genetic transformation of Citrus protoplasts and regeneration of transgenic plants. Plant Sci. 69, 199–206. 10.1016/0168-9452(90)90118-8

[B173] Vaucheret H. Fagard M. (2001). Transcriptional gene silencing in plants: targets, inducers and regulators. Trends Genet. 17, 29–35. 10.1016/S0168-9525(00)02166-1 11163919

[B174] Vaucheret H. Béclin C. Fagard M. (2001). Post-transcriptional gene silencing in plants. J. Cell Sci. 114, 3083–3091. 11590235 10.1242/jcs.114.17.3083

[B175] Viss W. J. Pitrak J. Humann J. Cook M. Driver J. Ream W. (2003). Crown-gall-resistant transgenic apple trees that silence *Agrobacterium tumefaciens* oncogenes. Mol. Breed. 12, 283–295. 10.1023/B:MOLB.0000006805.76717.08

[B176] Voinnet O. (2008). Post-transcriptional RNA silencing in plant-microbe interactions: a touch of robustness and versatility. Curr. Opin. Plant Biol. 11, 464–470. 10.1016/j.pbi.2008.04.006 18583181

[B177] Voinnet O. (2009). Origin, biogenesis, and activity of plant microRNAs. Cell 136, 669–687. 10.1016/j.cell.2009.01.046 19239888

[B178] Waltz E. (2011). Cisgenic crop exemption. Nat. Biotechnol. 29:677 10.1038/nbt0811-677b21822231

[B179] Waltz E. (2015). Non-browning GM apple cleared for market. Nat. Biotechnol. 33, 326–327. 10.1038/nbt0415-326c 25850045

[B180] Wang H. Alburquerque N. Burgos L. Petri C. (2011). Adventitious shoot regeneration from hypocotyl slices of mature apricot (*Prunus armeniaca* L.) seeds: a feasible alternative for apricot genetic engineering. Sci. Hortic. 128, 457–464. 10.1016/j.scienta.2011.02.020

[B181] Welander M. Zhu L. H. (2000). The rooting ability of *rolB* transformed clones of the apple rootstock M26 and its relation to gene expression. Acta Hortic. 521, 133–138. 10.17660/ActaHortic.2000.521.14

[B182] Wood A. J. Lo T. W. Zeitler B. Pickle C. S. Ralston E. J. Lee A. H.. (2011). Targeted genome editing across species using ZFNs and TALENs. Science 333, 307–307. 10.1126/science.1207773 21700836 PMC3489282

[B183] Wurdig J. Flachowsky H. Sab A. Peil A. Hanke M. (2015). Improving resistance of different apple cultivars using the *Rvi6* scab resistance gene in a cisgenic approach based on the FLP/*FRT* recombinase system. Mol. Breed. 35:95 10.1007/s11032-015-0291-8

[B184] Xie Z. Johansen L. K. Gustafson A. M. Kasschau K. D. Lellis A. D. Zilberman D.. (2004). Genetic and functional diversification of small RNA pathways in plants. PLoS Biol. 2:e104. 10.1371/journal.pbio.0020104 15024409 PMC350667

[B185] Zhao D. Song G. Q. (2014). Rootstock-to-scion transfer of transgene-derived small interfering RNAs and their effect on virus resistance in non-transgenic sweet cherry. Plant Biotechnol. J. 12, 1319–1328. 10.1111/pbi.12243 25132092

[B186] Zhao K. Zhang F. Yang Y. Ma Y. Liu Y. Li H. (2016). Modification of plant height via RNAi suppression of MdGA20-ox gene expression in apple. J. Am. Soc. Hortic. Sci. 141, 242–248.

[B187] Zhu C. Sanahuja G. Yuan D. Farré G. Arjó G. Berman J.. (2013). Biofortification of plants with altered antioxidant content and composition: genetic engineering strategies. Plant Biotechnol. J. 11, 129–141. 10.1111/j.1467-7652.2012.00740.x 22970850

[B188] Zhu T. Mettenburg K. Peterson D. J. Tagliani L. Baszczynski C. L. (2000). Engineering herbicide-resistant maize using chimeric RNA/DNA oligonucleotides. Nat. Biotechnol. 18, 555–558. 10.1038/75435 10802626

